# Introgressive hybridization and the evolutionary history of the herring gull complex revealed by mitochondrial and nuclear DNA

**DOI:** 10.1186/1471-2148-10-348

**Published:** 2010-11-11

**Authors:** Viviane Sternkopf, Dorit Liebers-Helbig, Markus S Ritz, Jun Zhang, Andreas J Helbig, Peter de Knijff

**Affiliations:** 1 German Oceanographic Museum, Katharinenberg 14-20, D-18439 Stralsund, Germany; 2 University of Jena, Institute of Ecology, Dornburger Str. 159, D-07743 Jena, Germany; 3 Department of Radiology, The University of Chicago, Chicago, Illinois, USA; 4 MGC-Department of Human and Clinical Genetics, Leiden University Medical Center, P.O. Box 9503, NL-2300 RA Leiden, The Netherlands

## Abstract

**Background:**

Based on extensive mitochondrial DNA (mtDNA) sequence data, we previously showed that the model of speciation among species of herring gull (*Larus argentatus*) complex was not that of a ring species, but most likely due more complex speciation scenario's. We also found that two species, herring gull and glaucous gull (*L. hyperboreus*) displayed an unexpected biphyletic distribution of their mtDNA haplotypes. It was evident that mtDNA sequence data alone were far from sufficient to obtain a more accurate and detailed insight into the demographic processes that underlie speciation of this complex, and that extensive autosomal genetic analysis was warranted.

**Results:**

For this reason, the present study focuses on the reconstruction of the phylogeographic history of a limited number of gull species by means of a combined approach of mtDNA sequence data and 230 autosomal amplified fragment length polymorphism (AFLP) loci. At the species level, the mtDNA and AFLP genetic data were largely congruent. Not only for *argentatus *and *hyperboreus*, but also among a third species, great black-backed gull (*L. marinus*) we observed two distinct groups of mtDNA sequence haplotypes. Based on the AFLP data we were also able to detect distinct genetic subgroups among the various *argentatus*, *hyperboreus*, and *marinus *populations, supporting our initial hypothesis that complex demographic scenario's underlie speciation in the herring gull complex.

**Conclusions:**

We present evidence that for each of these three biphyletic gull species, extensive mtDNA introgression could have taken place among the various geographically distinct subpopulations, or even among current species. Moreover, based on a large number of autosomal AFLP loci, we found evidence for distinct and complex demographic scenario's for each of the three species we studied. A more refined insight into the exact phylogeographic history within the herring gull complex is still impossible, and requires detailed autosomal sequence information, a topic of our future studies.

## Background

For speciation, the divergence of an ancestral population into two reproductively isolated sister taxa requires genetic differentiation of at least those loci involved in reproductive (sexual) functions [1]. With ongoing gene flow this process will be delayed and it is unclear to what extent gene flow must be reduced - or is still allowed - in order for speciation to be "complete" [2,3]. It was Ernst Mayr [4], who proposed that reproductive isolation could evolve through 'isolation-by-distance', i.e. with continuous gene flow, when peripheral populations meet after expanding around a large, uninhabitable area. This specific speciation model was later termed the 'ring species' model [5]. Geographic overlap between taxa that are elsewhere connected through interbreeding populations is an essential element of this model, because it is ongoing gene flow that distinguishes ring species from cases of allopatric speciation that happen to be arranged in a roughly circular fashion [6].

**Figure 1:**
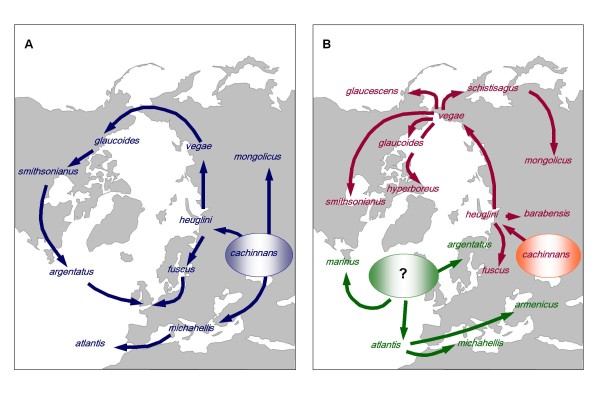
**Two contrasting hypotheses about the differentiation and colonization history of the herring gull complex**. Large ovals show hypothetical ancestral refugia. Arrows indicate inferred colonization routes. **(A) **Mayr's model [4], proposed that herring gulls originated in the Aralo-Caspian region (*cachinnans*), from where gulls spread in three directions (1) west via the Mediterranean into the Atlantic giving rise to Mediterranean (*michahellis*) and Atlantic (*atlantis*) yellow-legged gulls; (2) east toward Inner Asia giving rise to Mongolian gull (*mongolicus*) and (3) north to the Arctic Ocean (*heuglini*). Along the north Eurasian coasts, the ancestral population expanded into two opposite directions: (a) west across Scandinavia towards Britain and Iceland differentiating into dark-mantled lesser black-backed gulls (*fuscus*), and (b) east all the way to the North Pacific, giving rise to progressively paler-mantled forms *vegae *(eastern Siberia), and into North America (*glaucoides *and *smithsonianus*). Mayr proposed that, following the last Glacial Maximum, North American herring gulls (*smithsonianus*) subsequently crossed the North Atlantic and invaded Europe, where they gave rise to the pale-mantled European herring gull (*argentatus*) that now overlaps with the dark-mantled lesser black-backed gulls (*fuscus*) [4,9]. Note that Mayr did not include *marinus *and *hyperboreus *in his original model. **(B) **Alternative model based on results of Liebers et al. [8]. Two ancient refugia are inferred. Taxa derived from Atlantic refugium are shown in green, those derived from Aralo-Caspian refugium in red. No invasion of herring gulls from North America to Europe occurred. *Marinus *developed reproductive isolation in allopatry (probably in north-eastern North America) before making secondary contact with North American *smithsonianus *and Eurasian *argentatus/fuscus*. Two separate colonisation events from the Atlantic into the Mediterranean led to the differentiation of *armenicus *and *michahellis*.

For a long time, the herring gull (*Larus argentatus*) complex was considered the classical example of a ring species. The herring gull complex comprises of more than 20 taxa of large gulls [7] occupying a circumpolar breeding range in the northern hemisphere. The various herring gull taxa differ in body size, in the darkness of their dorsal plumage, and in bare part colours. According to Mayr's model [4], herring gulls originated in the Aralo-Caspian region, from where gulls spread in a number of different directions (see Figure 1A and reference [8]). Mayr and others envisioned all taxa of the circumpolar chain to be connected by gene flow, while herring gulls and lesser black-backed gull (*L. fuscus*) in Europe, the hypothetical endpoints of the ring, have reached full reproductive isolation and now coexist as distinct species [4,9].

In contrast, we found strong evidence that the ring species model did not adequately describe the evolution of the herring gull group because, contrary to Mayr's [4] proposal, there was no overlap between the endpoints of a ring of interbreeding taxa ([8], Figure 1B). We identified two major only distantly related groups of mitochondrial DNA (mtDNA) sequence haplotypes - termed clade 1 and clade 2 [8]. Sympatric coexistence, e.g. between *argentatus *(not derived from *smithsonianus *as assumed by Mayer) and *fuscus *in Europe, occurred between taxa with clade 1 and clade 2 mtDNA haplotypes and was not due to "circular overlap", but to secondary sympatry between forms that diverged in allopatry. We also found that more taxa than originally suspected (and not included by Mayr) were full members of the species complex, e.g. great black-backed gull (*Larus marinus*), and glaucous gull (*L. hyperboreus*).

Unexpectedly, we observed two biphyletic taxa in the mtDNA haplotype network with *argentatus *showing no discrete geographic pattern in carrying clade 1 or clade 2 mtDNA haplotypes. In contrast, Nearctic breeding *hyperboreus *carried exclusively clade 2 haplotypes whereas Palearctic breeding *hyperboreus *carried only clade 1 haplotypes. We provisionally explained this by the combined effects of hybridization events and past gene flow episodes or incomplete lineage sorting of a polymorphic ancestral gene pool. Recently, Vigfussdottir et al. [10] tried to unravel the underlying processes among these two biphyletic gull species with a combined molecular approach (using mtDNA sequence haplotypes and autosomal microsatellite genotypes) from *argentatus *and *hyperboreus *breeding in Iceland and Greenland. They also found the Icelandic breeding *hyperboreus *to be closely related to other European breeding *hyperboreus*, whereas the Greenland breeding birds share their genetic information exclusively with Nearctic *hyperboreus*. Vigfussdottir, like Liebers et al. [8] found mtDNA haplotypes of both clades among Icelandic *argentatus *populations. Interestingly, they also observed a change over time with respect to mtDNA clade membership, with an increase of clade 2 haplotypes among more recently sampled *argentatus*. It is obvious from both studies [8,10] that there is a need for a much more detailed investigation of these the two biphyletic taxa based on many more autosomal polymorphic loci. For this reason, we used AFLP (amplified fragment length polymorphisms [11]) as autosomal nuclear markers in the present study. Previous studies have shown that AFLP is a good marker system for population genetics [12-15], reconstruction of shallow phylogenies [16-18], population assignment [19], and hybrid detection [20].

Much to our surprise, we discovered a third biphyletic member of the herring gull complex, the great black-backed gull (*Larus marinus*) in the early stages of this study. Palearctic *marinus *all carry a clade 1 mtDNA haplotypes whereas Nearctic *marinus *displayed a mix of clade 1 and clade 2 haplotypes.

Therefore, the present study focuses more specifically on the three taxa that were biphyletic in the mtDNA network, *argentatus*, *hyperboreus*, and *marinus*, in order to obtain a more detailed insight into the various demographic/introgression events that caused the present-day pattern of genetic variation. We also included samples of a number of closely related taxa, Mediterranean and Macaronesian yellow-legged gulls (*michahellis*, and *atlantis*), Caspian gull (*cachinnans*), North American herring gull (*smithsonianus*), and lesser black-backed gulls (*fuscus *and *graellsii*), all members of the herring gull complex [8].

## Methods

### Sampling, taxon designation, and sample selection

Blood and tissue samples were taken from adults or unrelated chicks, almost exclusively from breeding colonies. The exception being five non-breeding *hyperboreus *sampled on the Faroe Islands during the winter. Taxon designations were based on phenotype of breeding adults and on geographic location. In this paper we describe results from a mixture of currently recognised species and subspecies that all clearly belong to the herring gull (*Larus argentatus*) species assemblage. These include (i) herring gulls breeding in the Palearctic (European herring gull, *L. argentatus*) with subspecies *argentatus*, and *argenteus*, (ii) herring gulls breeding in the Nearctic (American herring gull, *L. smithsonianus*), (iii) yellow-legged gulls from the Atlantic (*L. michahellis atlantis*) and the Mediterranean (*L. m. michahellis*), (iv) Caspian gull (*L. cachinnans*), (v) lesser black-backed gull (*L. fuscus*) with subspecies *fuscus *and *graellsii*, (vi) glaucous gull (*L. hyperboreus*) from its Nearctic and Palearctic breeding range, and (vii) great black-backed gull (*L. marinus*), also from its Nearctic and Palearctic breeding range. Throughout this manuscript we prefer to indicate all taxa by their terminal taxon names, because, although some are clearly separable phenotypically and/or geographically, others are not and their exact taxonomic position is far from unanimously defined.

Our sampling strategy mainly focussed on *argentatus*, *marinus*, and *hyperboreus*. A total of 377 *argentatus *individuals from 16 different European colonies were initially screened for their mtDNA *hypervariable region 1 *(*HVR1*) profile (Additional File 1 and Additional File 2). Of these, we selected a subset of 109 birds based on their *HVR1 *defined clade 1 or clade 2 memberships (Table 1). For each colony we tried to select an equal number of birds with clade 1 and clade 2. For two colonies, WSA and NET this was not possible. These 109 selected birds were further analysed by means of AFLP and mtDNA *cytochrome B *(*cytB*) sequencing (see below).

**Table 1:** Taxonomic designation, sample size, geographic origin, and colony abbreviation of gull populations used in this study

taxon	n	geographic origin	abbreviation	geographic coordinates	collected by
***argentatus***	10	Russia, White Sea	WSA	67°09'N,32°23'E	I. Charitonova, A. Filchagov
	6	Norway, Tromso	NNO	69°40'N,19°00'E	R. R. Snell
	6	Finland, Lake-Saimaa	FIN	61°16'N,28°15'E	R. Juvatse
	6	Estonia, Matsalu	EST	58°46'N,23°44'E	R. Juvaste
	2	Poland, Wloclawek	POL	52°39'N,19°05'E	M. Zielinski
	4	Germany, Hiddensee	MVP	54°20'N,13°10'E	R. Barth, A. J. Helbig
	4	Denmark, Lindholm	DEN	55°43'N,11°43'E	K. T. Pederson
	4	Sweden, NW Skane	SSW	56°27'N,12°34'E	K. Bengtson
	6	Norway, Vest-Agder	SNO	58°10'N,06°40'E	T. O. Hansen
	
***argenteus***	18	Iceland, Skruder	ICE	65°00'N,13°20'W	R. R. Snell
	10	Iceland, Karlsskali	ICW	65°00'N,13°20'W	R. R. Snell
	4	Denmark, Faroe Islands	FAR	62°20'N,07°20'W	R. R. Snell
	6	England, Isle of May	ENG	56°11'N,02°33'W	B. H. Bailey, M.Harris
	8	France, Finistere	FRA	48°40'N,03°20'W	R. R. Snell
	11	Netherlands, Maasvlakte	NET	51°56'N,04°28'E	F. Cottaar, N v. Swelm
	4	Germany, Helgoland	HGL	54°11'N,07°54'E	N. Robert

***hyperboreus***	8	Russia, Novaja Semlja	NOS	73°15'N,56°01'E	A. J. Helbig
**Eurasia**	10	Svalbard, Longyearbyen	SVA	78°13'N,15°20'E	R. R. Snell
	5	Denmark, Faroe Islands	FAR	61°35'N,05°00'W	J. K. Jensen
	9	Iceland, Bjarnhafnarfjall	ICB	65°00'N,23°00'W	R. R. Snell
	
***hyperboreus***	17	Canada, NWT, Baffin Island	BAF	69°00'N,68°00'W	R. R. Snell
**N. Am.**	7	USA, Alaska, Yukon	ALY	62°25'N,165°31'W	A. J. Baker
	9	USA, NW Alaska	ALA	71°11'N,163°51'W	J. A. Gerwin
	2	USA, Washington	WAS	47°20'N,120°05'W	R. Chandler, S. Rohwer

***marinus***	11	Denmark, Katholm, Jylland	KAT	55°43'N,11°43'E	E. Fritze, K. T. Pedersen
**Europe**	2	Netherlands, Maasvlakte	NET	51°56'N,04°28'E	F. Cottaar, N v. Swelm
	2	France, Finistere	FRA	48°40'N,03°20'W	R. R. Snell
	15	Denmark, Faroe Islands	FAR	61°35'N,05°00'W	J. K. Jensen
	2	Iceland, Skruder	ICE	65°00'N,13°20'W	R. R. Snell
	
***marinus***	3	Canada, Newfoundland	NFL	47°18'N,52°48'W	A. J. Baker
**N. Am**.	11	Canada, Bay of Fundy	FUN	45°12'N,86°09'W	C. Pekarik
	18	Canada, Lake Ontario	ONT	43°53'N,76°23'W	C. Pekarik

***atlantis***	3	Portugal, Berlenga Islands		39°24'N,09°30'W	M.v. Leeuwen, L. Moreis
	5	Morocco, Essaouria		31°29'N,09°45'W	M.v. Leeuwen, N. v. Swelm
	5	Portugal, Island of Madeira		32°52'N,17°10'W	M.v. Leeuwen, N. v. Swelm
	
***michahellis***	5	Spain, Gibraltar		36°08'N,05°21'W	M.v. Leeuwen, N. v. Swelm
	2	France, Alsace		48°10'N,08°00'W	M. Boschert
	4	Italy, Capraia Island		43°03'N,09°48'W	N. Baccetti
	2	Malta, Filfla Island		35°57'N,14°26'W	J. Sultana, C. Gauci
	5	Greece, Island of Crete		35°10'N,25°50'W	A. J. Helbig

***cachinnans***	6	Rumania, Danube Delta		44°30'N,28°30'E	R. Klein, A. Buchheim
	4	Ukraine, Odessa		46°20'N,32°30'E	A. Rudenko, N. v. Swelm
	7	Ukraine, Azov' Black Sea		44°30'N,28°30'E	V. Dierschke, D. Liebers
	16	Russia, N Caspian Sea		45°00'N,48°20'E	T. Tennhardt, D. Liebers

***fuscus***	17	Finland, Lake Saimaa		61°16'N,28°15'E	R. Juvaste
***graellsii***	4	Denmark, Faroe Islands		61°35'N,05°00'W	E. Fritze
	8	Iceland		64°09'N,21°57'W	A. Sigfusson

***smithsonianus***	6	Canada, Lake Ontario	ONT	45°20'N,80°02'W	D. Liebers
	15	Canada, New Brunsewick	NBR	47°51'N,64°33'W	R. R. Snell
	9	Canada, Prince Edward Island	PEI	46°10'N,63°30'W	R. R. Snell
	5	USA, Alaska, Fairbanks	ALF	64°50'N,147°10'W	R. R. Snell

Indicated are the commonly used taxonomic names, sample sizes, geographic origins, and sample collectors for all gull populations used in this study. The colony abbreviations correspond with those in figures 3, 4, and 5.

For *marinus *we screened 32 individuals from five European colonies and 32 individuals from three eastern North American colonies (Table 1). For *hyperboreus *we screened 32 birds from four Eurasian colonies and 35 birds from four North American colonies (Table 1). In addition, we also analyzed *smithsonianus *(35 individuals from four North American colonies), *fuscus*+*graellsii *(n = 30), *michahellis*+*atlantis *(n = 31), and *cachinnans *(n = 33), all for the same *HVR1*, *cytB *and AFLP profiles. Details of geographic origin and sample sizes are given in Table 1. Voucher material has been deposited at the German Oceanographic Museum in Stralsund.

### Laboratory methods

#### Mitochondrial DNA sequencing

Laboratory protocols for DNA extraction, amplification and sequencing of mtDNA *cytB *gene and *HVR1 *have been described in detail elsewhere [8,10].

#### AFLP genotyping

The AFLP-protocols followed Vos et al. [11]. Briefly, total genomic DNA (300 ng) was restricted over night at 37°C with 3 units each of *Eco *RI and *Mse *I (by Fermentas), after which a ligation mix containing 0,5 pmol/μl E-adaptor and 5 pmol/μl M-adaptor and 0,5 units of T4 DNA-ligase was added and incubated at 37°C for 4 hours. A preselected amplification was performed with an additional C at the 3'-end of the *Eco *RI-primer and the *Mse *I-primer (Table 2).

**Table 2:** Primer sequences and names used for screening and scoring of AFLP fragments

EcoR - primer sequences	
**for pre-amplification:**	5'-GACTGCGTACCAATTC-**C**-3'

**for sequencing**	5'-GACTGCGTACCAATTC-**Cxx**-3'

**ending:**	**primer name:**

CAA	EcoC1

CAC	EcoC2

CAT	EcoC3

CCA	EcoC4

CCC	EcoC5

CCT	EcoC6

CGC	EcoC7

CGT	EcoC8

CTA	EcoC9

CTT	EcoC10

**Mse - primer sequences**	

**for pre-amplification:**	5'-GATGAGTCCTGAGTAA-**C**-3

**for sequencing**	5'-GATGAGTCCTGAGTAA-**Cxx**-3

**ending:**	**primer name:**

CAC	Mse1

CAG	Mse2

CAT	Mse3

CCA	Mse4

CCT	Mse5

CGA	Mse6

CGT	Mse7

CTA	Mse8

CTG	Mse9

CTT	Mse10

**Selected 17 primer-pairs**	

**Primer combinations used**	**number of detected variable loci**

EcoC1 Mse8	13

EcoC1 Mse10	18

EcoC2 Mse4	6

EcoC2 Mse5	5

EcoC2 Mse6	20

EcoC2 Mse7	5

EcoC2 Mse8	12

EcoC3 Mse1	17

EcoC3 Mse2	13

EcoC3 Mse4	9

EcoC3 Mse5	10

EcoC3 Mse10	22

EcoC4 Mse1	17

EcoC4 Mse7	27

EcoC5 Mse1	14

EcoC5 Mse3	11

EcoC7 Mse1	11

This table lists the used EcoR1 and Mse1 core-primer and their triplet endings for the pre- amplification and sequencing step. For the pre-amplification only one primer pair was chosen (EcoR1+C/Mse1+C). The full primer sequences are indicated. In order to find the best sequence primer pairs (showing the highest level of variable loci) for final analyses we screened all possible 100 combinations of triplet-endings. We selected those 19 combinations that showed more than five variable loci when screening a set of six *cachinnans *and six *michahellis *(the two most distinct taxa in the mtDNA shown in reference [8]. Of these, 2 combinations were subsequently removed because they were not sufficiently variable among our full set of gulls. The remaining final set of 17 primer pairs are given in this table, together with their number of variable loci resulting in the whole dataset.

The product of the preselective amplification was diluted 20 times and used in a series of selective PCR amplifications aimed at testing 100 combinations of selective primers, each containing three additional nucleotides at the 3'-end (including the C of the preselective PCR). The fragments were separated in 6% polyacrylamid gels and detected by fluorescein labelled *Eco *RI-primer on a Li-Cor DNA Sequencer (Long Reader 4200a).

### Analysis of mitochondrial DNA

All *cytB *sequences (1143 bp) were aligned without gaps and contained no stop codons. Alignment of *HVR1 *sequences (391 - 430 bp) required the insertion of single gaps at three positions, which were deleted prior to further analysis.

To provisionally explore the mitochondrial population structure of all samples (including the full set of 377 *argentatus HVR1 *sequences, see Additional File 1) throughout Europe we computed a UPGMA-tree using the average distance BLOSUM62 [21] routine in JALVIEW [22] based on the *HVR1 *sequence only (Additional File 2). Based on this tree, all samples were assigned to either clade 1 or clade 2.

A subset of 109 *argentatus *individuals (Table 1) was selected for more detailed analysis, based on their mtDNA clade 1 or clade 2 membership. For all colonies except those from the White Sea (WSA) and the Netherlands (NET) we were able to choose equal numbers of birds from mitochondrial clade 1 and clade 2. From these 109 *argentatus*, together with the 260 individuals from the other six taxa, a complete concatenated mtDNA sequence alignment was constructed, containing a total of 369 *cytB *and *HVR1 *sequences (see Additional File 3).

**Figure 2:**
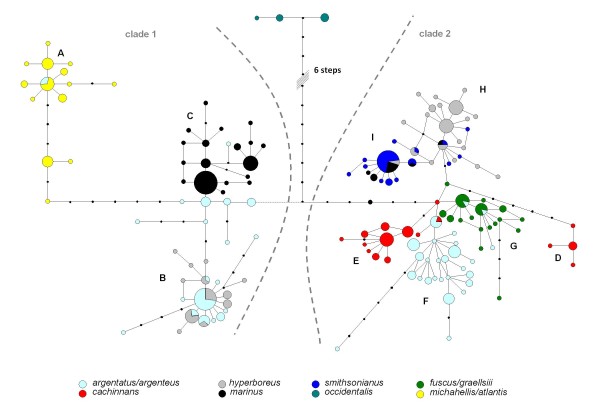
**Median-joining network of mtDNA haplotypes among herring gull taxa**. Median-joining network of 368 concatenated *cytB *and *HVR1 *mtDNA sequence haplotypes (see Additional file 2) of all gull taxa included in this study. Distinct haplotype groups are labelled A-I. The network was rooted by the inclusion of previously published [8]*occidentalis *sequences. The network shows three biphyletic taxa of which individuals either carry clade 1 or clade 2 haplotypes: The European herring gull (*argentatus/argenteus *- pale blue) in haplogroup B, F and G; the glaucous gull (*hyperboreus *- grey) in group B, H and I; and the black-backed gull (*marinus *- black) in haplogroup C, I and H.

A median-joining network was constructed using NETWORK v. 4502 [23]. Variable sites were differentially weighted reciprocally according to their site-specific mutation rate in the total network. Rooting of the network was done by the use of previously published [8] Western gull (*L. occidentalis*) sequences. The final network figure was made using a combination of NETWORK and NETWORK PUBLISHER (Figure 2).

### Analysis of AFLP

The initial AFLP selection was performed by screening the two most distinct taxa in the mitochondrial network, *cachinnans *(n = 6) and *michahellis *(n = 6) for all combinations of 10 EcoRI and 10 MseI primers, resulting in different 100 primer combinations (Table 2). We selected those primer combinations that showed more than five variable loci. As a result of this, 19 primer combinations were chosen for the present study. Of the 19 primer combinations only the products of 17 combinations could be score reliably in the full dataset. AFLP fragments were coded as '1' (presence of a fragment) or '0' (absence of a fragment). We further reduced the total dataset to only those primer combinations that resulted in scores among all individuals tested (this reduction also included the sex-specific AFLP loci). As a result of this, the total AFLP dataset used for this study consisted of the 1/0 scores of 230 loci among 369 individuals (available upon request).

In order to identify AFLP loci that could be used to better distinguish all distinct seven taxa, locus specific Fst-values were calculated in TFPGA v.1.3 [24]. Using a minimum threshold of 0.2, we identified among all 230 loci, 43 most discriminating AFLP loci. TFPGA was also used to estimate unbiased heterozygosities averaged across all loci for each population separately (not shown).

**Figure 3:**
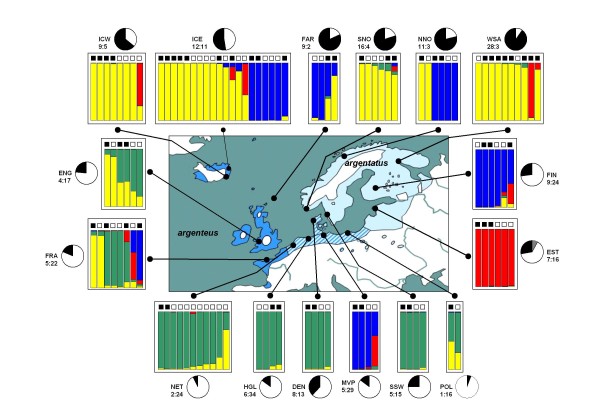
**Geographic distribution, mtDNA haplotype distribution, and autosomal AFLP based admixture proportions of European herring gull (L. argentatus) populations**. The geographical distribution of the two European herring gull subspecies are indicated in solid dark blue (western subspecies *argenteus*), and light blue (north-eastern subspecies *argentatus*). Their geographical overlap (striated area) is also shown. Two mtDNA *HVR1 *sequence haplotype groups were identified (Additional file 1, Figure 2, Additional file 2, and reference [8]): clade 1 and clade 2. For each of the 16 herring gull colonies the black pie chart area reflects the relative frequency of individuals with clade 1 mtDNA haplotypes. Similarly, the white area corresponds to clade 2. Also, the exact number of individuals with clade 1 (left) and clade 2 (right) haplotypes and abbreviated colony names (Table 1) are indicated. Two *cachinnans *haplotypes, shown in grey, are indicated in the Estonian (EST) colony. Clade 1 mtDNA haplotypes are more frequent among northern colonies, whereas clade 2 mtDNA haplotypes are more frequent among southern colonies. There is less congruence between mtDNA haplotype distribution and sub-species: northern Icelandic *argenteus *displays predominantly clade 1 haplotypes. Eastern Finnish *argentatus *displays predominantly clade 2 mtDNA haplotypes. The boxed areas define, for each colony, those individuals that we used for the analyses of 230 AFLP loci and *cytB *sequencing. AFLP genotypes were analysed by STRUCTURE and revealed the presence of four distinct ancestral populations among present day *argentatus*, here shown with yellow, green, blue, and red. Each individual is represented by a (multi) coloured bar, on top of which there is a black-filled square (clade 1 membership), or a white square (clade 2 membership). The proportion of each colour within a single bar indicates the relative contribution of one of these four ancestral populations to the genome of that individual gull.

**Figure 4:**
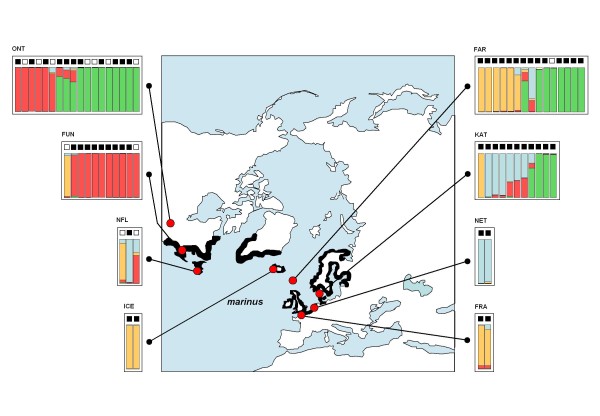
**Geographic distribution, mtDNA haplotype distribution, and autosomal AFLP based admixture proportions of great black-backed gull (L. marinus) populations**. The breeding range (black), and sampling locations (red dots) of *marinus*. For each of the eight colonies a boxed area contains individuals that we used for the analyses of 230 AFLP loci and *HVR1 *and *cytB *mtDNA sequencing. Colony name abbreviations above the top-left corner of each box correspond with those in Table 1. Genotypes were analysed by STRUCTURE and revealed the presence of four distinct ancestral populations among present day *marinus*. In this figure these are represented by four different colours (pale red, pale blue, pale green, and pale orange). Each individual is represented by a single (multi) coloured bar, on top of which there is either a black filled square (indicating that this individual displayed an mtDNA clade 1 haplotype), or a white square (representing clade 2 mtDNA haplotypes). The proportion of each colour within a single bar indicates the relative contribution of one of these four ancestral populations to the genome of that gull. For instance, the top left panel represents 18 *marinus *from a Nearctic population (ONT). Of these, 11 displayed a clade 1 mtDNA haplotype (black squares above the coloured bars), whereas seven displayed a clade 2 mtDNA haplotype (white squares). Among the 18 birds, the leftmost five display a 100% fixed autosomal contribution of the ancestral population coded by pale red to their genomes. The rightmost nine individuals show 100% fixed contribution of another (pale green) ancestral population. There are also four individuals with various degrees of admixed contributions of two or more ancestral populations.

**Figure 5:**
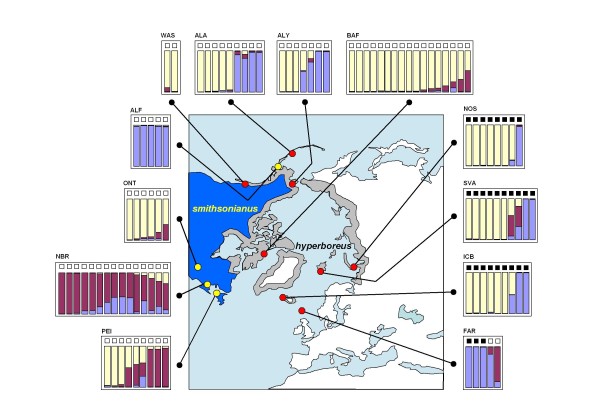
**Geographic distribution, mtDNA haplotype distribution, and autosomal AFLP based admixture proportions of glaucous gull (L. hyperboreus) and American herring gull (L. smithsonianus) populations**. The breeding ranges (blue for *smithsonianus*, grey for *hyperboreus*), and sampling locations (yellow for *smithsonianus*, red for *hyperboreus*). For each of the 12 colonies a boxed area contains all individuals that we used for the analyses of 230 AFLP loci and *HVR1 *and *cytB *mtDNA sequencing. Colony name abbreviations above the top-left corner of each box correspond with those in Table 1. Genotypes were analysed by STRUCTURE and revealed the presence of three distinct ancestral populations among present day *smithsonianus *and *hyperboreus*. In this figure these are represented by three different colours (pale yellow, pale blue, and red). Each individual is represented by a single (multi) coloured bar, on top of which there is either black-filled square (indicating that this individual displayed an mtDNA clade 1 haplotype), or a white square (representing clade 2 mtDNA haplotypes). The proportion of each colour within a single bar indicates the relative contribution of one of these three ancestral populations to the genome of that gull. For instance, the bottom left panel represents nine *smithsonianus *from a Nearctic population (PEI). They all display a clade 2 mtDNA haplotype (white squares). Among the nine birds, the leftmost three display a nearly 100% fixed autosomal contribution of the ancestral population coded by pale yellow to their genomes. The rightmost three individuals show a near 100% fixed contribution of another (red) ancestral population. The remaining three individuals display various degrees of admixed contributions of the three ancestral populations.

**Figure 6:**
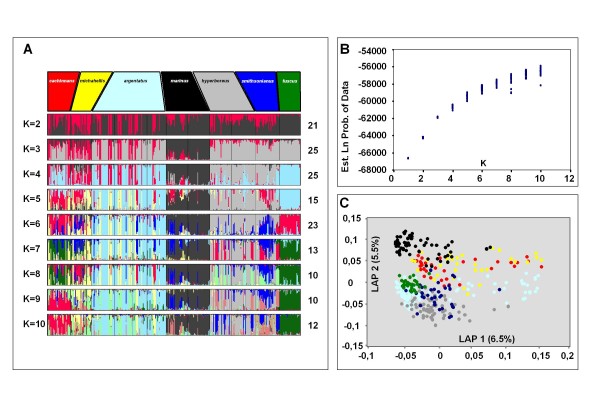
**Results of STRUCTURE and LAPEA analyses for all 230 AFLP loci**. A STRUCTURE and Laplacean Eigenvector analysis (LAPEA) was performed on 369 gulls, representing seven distinct species (taxon names indicated in the top row boxes in panel A). **(A)**. STRUCTURE was used to explore values of K = 1 to K = 10. For each K 25 independent runs were performed. Consistency among runs was explored by the combined use of Structure Harvester, CLUMPP, and DISTRUCT. For each value of K, the most frequent mode is displayed, with the number of independent runs with this mode indicated to the right of each multicoloured panel. Individuals are depicted as vertical bars segmented in their calculated membership of each of the various clusters (reflecting ancestral populations). Each colour reflects the estimated relative contribution of each of the populations to that individual's AFLP-based genome. Individuals are *posteriori *sorted according to their taxon definition. K = 6 is the most optimal overall number of clusters in the total dataset, with 23 out of 25 runs with exactly the same mode of differentiation. At K = 7 two clear modes were visible, with the one shown here being most frequent (13 out of 25), but failed to differentiate *fuscus *from *cachinnans*. The other (12 out of 25, not shown) did represent a mode that could differentiate all seven taxa. **(B)**. A graph showing for each of the 25 replicate runs for K = 1 to k = 10, the Ln probability of the data estimated by STRUCTURE. **(C)**. LAPEA of 230 AFLP loci of all 369 gulls from seven different taxa. Shown are the first two Eigenvectors and the percentage of the total variance they explain. Each dot represents a single individual and its colour corresponds with those of the seven taxa in panel (A). None of the seven taxa could be clearly differentiated.

**Figure 7:**
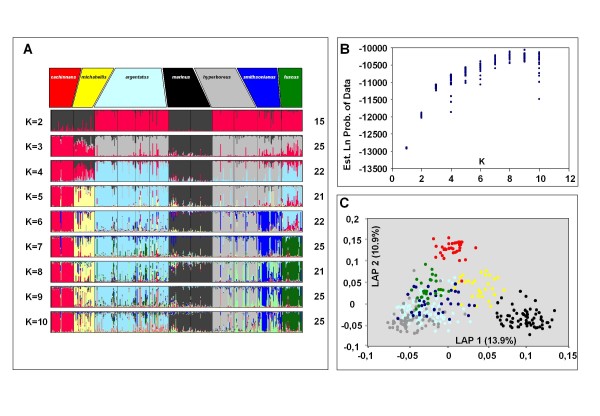
**Results of STRUCTURE and LAPEA analyses for 43 selected AFLP loci**. A STRUCTURE and Laplacean Eigenvector analysis (LAPEA) was performed on 369 gulls, representing seven distinct species (taxon names indicated in the top row boxes in panel A). Based on Fst calculation, 43 AFLP loci (Fst> 0.2 or higher according to a TFPGA analysis on the complete dataset) were selected for these analyses. **(A)**. STRUCTURE was used to explore values of K = 1 to K = 10. For each K 25 independent runs were performed. Consistency among runs was explored by the combined use of Structure Harvester, CLUMPP, and DISTRUCT. For each value of K, the most frequent mode is displayed, with the number of independent runs with this mode indicated to the right of each multicoloured panel. Individuals are depicted as vertical bars segmented in their calculated membership of each of the various clusters (reflecting ancestral populations). Each colour reflects the estimated relative contribution of each of the populations to that individual's AFLP-based genome. Individuals are *posteriori *sorted according to their taxon definition. K = 7 is the most optimal overall number of clusters in the total dataset, with all 25 runs with exactly the same mode of differentiation. This mode enabled the differentiation of all seven taxa. **(B)**. A graph showing for each of the 25 replicate runs for K = 1 to K = 10, the Ln probability of the data estimated by STRUCTURE. **(C)**. LAPEA of 43 AFLP loci of all 369 gulls from seven different taxa. Shown are the first two Eigenvectors and the percentage of the total variance they explain. Each dot represents a single individual and its colour corresponds with those of the seven taxa in panel (A). Note that three taxa, (*cachinnans*, *michahellis*, and *marinus*) clearly cluster in distinct groups. The other taxa remain weakly differentiated.

Population structure was explored in two ways. First we used the program STRUCTURE 2.2.3 [25,26] assuming admixture and correlated allele frequencies and a recessive genotype mode. For each run, the number of clusters, K, needs to be specified a-priori and we used values in the range of 1-10. For the burn-in-period, the number of iterations used was 20.000. For MCMC replications we used 10.000 iterations. For each value of K we repeated STRUCTURE analysis 25 times in order to explore consistency. For additional analyses within three species we used the same settings. The STRUCTURE output files were first processed using STRUCTURE HARVESTER v0.3 [27]. This produces an output consisting of a series of files, including graphical files representing, per K and per repeated run, the estimated Ln probability of each run, and three other Ln based estimates that allow the selection of the most optimal value for K [28]. Also included are files that can be used as input file in CLUMPP [29]. Subsequently CLUMPP produces output files that can be used as input in DISTRUCT [30]. We used CLUMPP to estimate, per K, the number of identical repeated runs. DISTRUCT was used to synchronize colour coding per ancestral population among repeated runs per K. Samples were analysed without any prior population information, but are sorted by their sampling population once STRUCTURE is completed. The STRUCTURE output graphs in Figures 3, 4, 5, 6, and 7 were produced using Microsoft Excel and Microsoft PowerPoint based on averaged raw STRUCTURE output tables.

Our second approach involved using Laplacian Eigenfunction analysis (LAPEA [31]). LAPEA, in analogy to e.g. principal components analysis (PCA), is a statistical tool one can use to achieve dimension reduction of highly complex sets of (genetic) data. A major advantage of LAPEA over PCA is that it compares each individual only to its close neighbours, rather than to all other individuals (where, here, closeness refers to genetic relatedness, not geographic distance). This makes LAPEA much less sensitive to distinct outliers, compared to PCA. As with PCA, LAPEA results can be visualized as simple two-dimensional dot-plots. In these plots, genetically similar individuals cluster more close together. Also in analogy to PCA, LAPEA results consist of more than two Eigenvectors and each of these explains part of the total observed variance. Here we only show the results of the first two vectors and the percentage of total variance they explain.

What LAPEA shares with STRUCTURE is that they both allow the analysis of large numbers of individuals for large numbers of genetic loci. Moreover, they are both based on comparing the genetic profiles of individuals without any prior classifier. Only after analyzing the samples, each individual can be labelled in retrospect.

### Web Resource

Accession numbers for all mtDNA sequences are listed in Additional file 1 and Additional file 3. The corresponding sequences can be found at:

GenBank, http://www.ncbi.nlm.nih.gov/GenBank/

Network software can be found at:

http://www.fluxus-engineering.com/sharenet.htm

Network Publisher can be purchased via:

http://www.fluxus-engineering.com/sharenet.htm

Structure Harvester can be found at: http://users.soe.ucsc.edu/~dearl/software/struct_harvest/

## Results

### Mitochondrial phylogeography

Based on the concatenated mtDNA-*HVR1 *and *cytB *sequences of 369 large white headed gull individuals (109 selected *argentatus *and 260 individuals of six other species; Table 1) a median joining network was drawn (Figure 2). As expected, the basic structure of this network is very similar to the one previously published by us [8]. In the complete network shown in Figure 2, two distinct groups (or clades) can be readily identified. Clade 1 consists of all *michahellis *and *atlantis*, part of *marinus*, part of *hyperboreus*, and part of *argentatus *individuals. Clade 2 contains the remaining *marinus*, *hyperboreus*, and *argentatus *individuals, in addition to all *cachinnans*, all *smithsonianus*, and all *fuscus *and *graellsii *individuals.

### Mitochondrial (sub)structure of *argentatus, marinus, and hyperboreus*

Based on the UPGMA-tree of mtDNA-*HVR1 *sequences (Additional File 2), each of the 377 *argentatus *individuals was assigned to either the mtDNA sequence haplotype defined clade 1 or clade 2. This clade composition, per each of the 16 different colonies, is illustrated as black-and-white pie charts in Figure 3. Among the more northern distributed colonies, the majority of individuals carry clade 1 haplotypes (black proportion), while clade 2 haplotypes (white proportion) are more frequent throughout the southern colonies.

Of the 32 European *marinus *individuals all but one (a bird from the Faroe Islands), display clade 1 mtDNA haplotypes, (here indicated for each individual separately by small black squares in Figure 4). Among the 32 North American *marinus *we find a more admixed distribution. The majority (n = 21) have clade 1 mtDNA haplotypes, while the remaining (n = 11) have clade 2 mtDNA haplotypes (indicated by white squares in Figure 4).

Among *hyperboreus *the picture is more clear. All but two out of 32 European *hyperboreus *(representing wintering birds from the Faroe Islands) have clade 1 mtDNA haplotypes (black squares in Figure 5). However, all 35 North American *hyperboreus *have clade 2 haplotypes (white squares in Figure 5).

### AFLP; Overall results

Of the original 19 AFLP primer combinations only the products of 17 combinations were used. Consequently, the resulting final AFLP dataset consisted of the 1/0 scores of 230 loci among 369 individuals (available upon request).

We explored the genetic population structure among all 369 individuals in two unbiased ways. First, we used a modified version of STRUCTURE that allows the analysis of recessive loci [25], and second, using Laplacian Eigenfunction analysis (LAPEA) [31]. By means of STRUCTURE, we attempted to assess whether or not the complete sampled dataset contained hitherto hidden substructure consisting of an unknown number of distinct (ancestral) genetic subgroups, or simply constituted one single panmictic population in Hardy Weinberg equilibrium. It is important to stress here, that by doing so we initially ignore species/subspecies designations and sampling locations of the individuals included in this analysis. This information is only used to group/cluster individuals once STRUCTURE analysis is completed. We performed a series of STRUCTURE runs varying between one and ten a-priori defined genetic subgroups or clusters (K = 1 to K = 10) using all 369 individuals and all 230 AFLP loci in order to detect the most optimal value of K in the total dataset. Selection of the optimal number of clusters was done by post processing all runs by means of STRUCTURE HARVESTER [27], allowing the combined use of visual inspection of the resulting plots, and methods proposed by Evanno [28]. For each K we performed 25 independent runs, and by means of the combined use of CLUMPP [29] and DISTRUCT [30], we could explore, summarize and visualize all STRUCTURE results (Figure 6A and 6B). Based on all 230 AFLP loci there was a clear optimum of K = 6, with 23 out of 25 runs showing nearly identical results (Figure 6A). It is remarkable that at K = 6 not all distinct taxa can be readily distinguished. Only *argentatus *(indicated by pale blue), *marinus *(black), and *hyperboreus *(grey) could be clearly distinguished. Distinct taxa such as *fuscus *and *cachinnans *were grouped into one (visualised by red) genetic cluster. We speculated that this could be due to a high number of non-informative AFLP loci in the total dataset and repeated the complete selection procedure using only those AFLP loci (n = 43) with an Fst value of 0.2 or higher according to a TFPGA analysis on the complete dataset (Figure 7A and 7B). This resulted in K = 7 being the optimal number of clusters, with all 25 runs resulting in near identical results (Figure 7a). These now gave consistent results with all seven taxa identified as genetically distinct subgroups, when using 43 polymorphic autosomal AFLP loci. Still, among all taxa, multiple individuals with autosomal genetic contributions typical for other taxa could be observed, a clear signal for relatively close genetic affinities and/or ongoing gene flow within this species complex.

Also by means of LAPEA we analysed the full set of 230 AFLP loci and the set of 43 AFLP loci selected because of their high among taxon Fst values (Figures 6C and 7C). We only show the graphical results based on the first two Laplacian Eigenvectors in both datasets. As with STRUCTURE, using all 230 loci, LAPEA only weakly discriminates between all taxa, whereas the 43 AFLP locus set resulted in a much more clear differentiation. Based on the 43 loci, *marinus*, *cachinnans*, and *michahellis *each group into distinct clusters, with the remaining taxa (*fuscus*, *argentatus*, *hyperboreus*, and *smithsonianus*) more weakly defined (Figure 7C). The difference in resolution between the two sets of loci is also reflected in the percentage of variance explained by LAPEA. For the 230 AFLP loci the first two Eigenvectors explained 11.9% (6.4% and 5.5% respectively), whereas for the set of 43 selected AFLP loci the first two Eigenvectors explained 24.8% (13.9% and 10.9% respectively).

### AFLP; herring gulls, *argentatus*

Using all 230 AFLP loci we analysed all 109 *argentatus *by means of STRUCTURE (Figure 3) and LAPEA (Additional File 4). For STRUCTURE, we explored a range of clusters (from K = 1 to K = 10). Again, for each K we performed 25 independent runs and all results were processed by means of STRUCTURE HARVESTER, CLUMPP, and DISTRUCT. We observed a clear optimum at K = 4 (results not shown) indicating that among present day *argentatus *we see the presence of at least four distinct (ancestral) genetic populations. We subsequently plotted, for each individual and each sampling site, the results of the STRUCTURE K = 4, and the mtDNA based clade 1/clade 2 designation (Figure 3). We observed a remarkable complex autosomal substructure among all 109 European *argentatus *individuals. In Figure 3 and Additional File 4, we indicated each of the contributions of these four ancestral populations by means of a unique colour. In analogy to the mtDNA sequence variation, also among AFLP loci we observed two genetic components with a clear north/south differentiation. One (indicated with yellow) is predominantly present among *argentatus *individuals from northern populations, with many individuals showing (close to) 100% of their autosomal gene pool comprised by this component. Another distinct genetic component (green) is predominantly present, with (nearly) fixed contributions) among *argentatus *from southern populations. However, there are also clear signals from two other ancestral populations or demographic events. One of these (the blue component) is present among individuals from all over the entire distribution of *argentatus *and was already notable at K = 2 (not shown) in exactly the same individuals and frequencies as shown in Figure 3 (with K = 4). This suggests that it could represent a signal of an important (perhaps early) demographic event. At K = 3 (not shown), the fourth (red) component became visible, also in exactly the same individuals as shown in Figure 3. Only at K = 4, the green and yellow components became visible as distinct genetic entities. This strongly suggests that these latter two components (green and yellow) reflect a more subtle (or recent) demographic event. There was no correlation between mtDNA clade membership and carrier status of any of the four autosomal genetic components (Chi-square test not significant, results not shown). To exclude the possibility that this marked genetic substructure among Eurasian herring gulls could be due to autosomal influences introduced by *hyperboreus*, a taxon we speculate (see below) to have expanded out of the Nearctic into the Palearctic relatively recently, we performed a STRUCTURE analysis using the AFLP genotypes of the combined group of *argentatus *(n = 109) and *hyperboreus *(n = 67) individuals. Since we were unable to detect any shared genetic component between these two taxa (results not shown), it is not very likely that the substructure among *argentatus *is due to *hyperboreus *influences.

These STRUCTURE results were independently confirmed by LAPEA analysis (Additional File 4). The first Eigenvector (explaining 12.3% of the total variance) clearly separates the two more ancient events/populations (blue and red) from the more recent contributions (green and yellow). The second Eigenvector (explaining 5.3% of the total variance) provides a strong contrast between blue and red, and also differentiates green from yellow. Note the seven individuals with clear intermediate positions between the three clusters. These individuals also show approximately equally admixed contributions by STRUCTURE. Only at higher levels (third and fourth Eigenvectors) green and yellow could be more clearly distinguished (results not shown). Alternatively, when only the green/yellow individuals were analysed separately, they could be distinguished at the first Eigenvector (results not shown). Taken together, STRUCTURE and LAPEA analyses both supported the presence of genetic signals of at least four distinct ancestral populations among present day *argentatus*, with, very likely, the most recent ones (still) enabling a differentiation between northern and southern *argentatus *populations.

### AFLP; great black-backed gulls, *marinus*

Also *marinus*, based on *2*30 autosomal AFLP loci, STRUCTURE (figure 4) and LAPEA (Additional File 5) demonstrated an unexpected autosomal complexity. Using STRUCTURE, we observed a distinct maximum at K = 4 ancestral populations. One of these (indicated with pale red) is predominantly present among Nearctic *marinus*, whereas the other three (pale green, pale blue and pale orange) are observed among all populations, although with varying frequencies and levels of admixture. As with *argentatus*, LAPEA results in *marinus *also corresponds with STRUCTURE in terms of the order of identification of each of the four distinct ancestral populations. Pale orange and pale green (clearly distinct by the first Laplacian Eigenvector) were also identified by STRUCTURE K = 2 and K = 3. Pale red and pale blue only became visible as distinct groups at STRUCTURE K = 4, and can not easily be separated by the first two Laplacian Eigenvectors (together explaining 16% of the total variance). It appears that the birds of the North American populations are more genetically homogeneous than in the European populations, which is reflected by their reduced unbiased heterozygosity (0.23) compared to European *marinus *(0.29, not shown).

### AFLP; glaucous gulls, *hyperboreus*

Because of their very close genetic affinities ([8], see also Figure 2), we analysed the AFLP data from Eurasian and North American *hyperboreus *together with another Nearctic species, *smithsonianus*. We again used STRUCTURE (Figure 5) and LAPEA (Additional File 6) to analyse the 230 AFLP loci. STRUCTURE revealed a clear optimum at K = 3, and this is confirmed by LAPEA. The first two Laplacian Eigenvectors (Additional File 6; explaining 12% of the total variance) were sufficient to distinguish between each of the three distinct ancestral populations indicated by STRUCTURE at K = 3 (Figure 5). Apart from the marked difference in mtDNA clade membership (see before), there is no clear autosomal difference between Nearctic and Palearctic *hyperboreus*. In both groups of populations we see the contributions of two ancestral populations (pale yellow and pale blue), with some admixture of a third component (red). This latter component is present in a very high frequency among two north-east American *smithsonianus *populations. A third *smithsonianus *population (from Lake Ontario) strongly resembles the majority of the *hyperboreus *populations (predominantly pale yellow with some red admixture), whereas the fourth *smithsonianus *population (from Fairbanks, Alaska) harbour individuals that are inseparable from many Nearctic *hyperboreus *(nearly fixed for the pale blue component, carrying clade 2 mtDNA haplotypes).

## Discussion

### Evolution of European *argentatus*

The European herring gull *L. argentatus *has long been considered a single species with many different subspecies and/or geographical variants [4,7,9]. We were able to reduce this complex picture into an assemblage including several distinct taxa (e.g. *argentatus *and *smithsonianus*) which were not each other's closest relatives [8]. Contrary to Mayr's [4] proposal, the herring gull assemblage did not represent a ring species model. We discovered that the current mitochondrial DNA genetic make-up of the *argentatus *showed clear signs of past hybridisation between birds derived from different ancestral refugia, although no definite geographic scenario could be reconstructed for this reticulation because of insufficient sampling in our previous study. Such a biphyletic representation in the mtDNA haplotype network (also evident in *hyperboreus *and *marinus*, see Figure 2 in this article) provided a striking illustration of how discrepancies could arise between a single gene tree (in this case mtDNA based) and a taxon phylogeny. The fact that some gull species, apparently due to complex past demographic events, contain highly divergent mtDNA haplotypes suggests that mitochondrial lineage sorting could have quite different and unpredictable outcomes.

In the present study we tried to obtain more clarification into the apparent north versus south genetic differentiation in *argentatus*. Based on a detailed mtDNA analysis among 377 European herring gulls from 16 different breeding colonies we now clearly demonstrate that the more northern breeding birds (based on geography considered to be nominate *argentatus *and/or *omissus*) display predominantly clade 1 mtDNA haplotypes, whereas southern breeding birds (of the subspecies *argenteus*) display typical clade 2 mtDNA haplotypes (see the pie charts in Figure 3). Using STRUCTURE, and independently confirmed by LAPEA, we found strong evidence for a complex autosomal genetic substructure among *argentatus *based on 230 AFLP loci (Figure 3, Additional File 4). This substructure, consisting of four distinct ancestral populations, is partly explained by a distinct northern (yellow in both figures) and a distinct southern (green in both figures) distribution. However, at the level of individual gulls these did not correlate with the two geographically distinct mtDNA clades. We also observed genetic signals of two other ancestral populations, probably reflecting more ancient demographic events (red and blue). This pattern of a number of distinct ancestral contributions among present day individuals resembles very much the distinct substructure pattern among globally dispersed human populations. Based on genome wide sets of genetic polymorphisms [32], human populations carry the signals of a number of marked demographic events, generally assumed to reflect the combined effects of genetic bottlenecks and substantial migration events, leading to a distinct clinal pattern of isolation by distance. The timing of this process is often reflected in the hierarchy by means of which distinct ancestral genetic populations was estimated by STRUCTURE. Among humans, the first marked event is nearly always visible at K = 2 (distinguishing African from non-African populations). If we assume that the patterns revealed by STRUCTURE and LAPEA among *argentatus *also reflects (in part) the temporal timing of a number of important demographic events, the most likely scenario explaining the genetic substructure among *argentatus *is the one whereby the blue component represents the first expansion of birds (carrying clade 1 mtDNA haplotypes, [8]) out of the original Aralo-Caspian refugium. The red autosomal component subsequently reflects the second expansion (carrying mtDNA clade 2 haplotypes) out of the same Aralo-Caspian refugium. A relatively more recent process (the last Glaciation?) could subsequently be responsible for the differentiation between discrete northern (yellow) and southern (green) *argentatus *populations, both involving birds that already harboured signals from the two more ancient events. The complex present day distribution of both the two mtDNA types as well as the four autosomal types among *argentatus *is most likely explained by an ongoing, and complex process of introgression while populations expand and contract [33].

### Colonization pattern of *marinus*

Our previous study did not support the traditional view of greater black-backed gulls (*L. marinus*) being a distinct outgroup relative to the herring gull complex, although *marinus *is fully reproductively isolated from all species it co-occurs with [8]. We suggested that *marinus *diverged in allopatry from the rest of clade 1. Our present study now also includes a substantial number of Nearctic breeding *marinus *(n = 32) in addition to 32 samples from Palearctic breeding colonies. Apart from a single individual from the Faroe Islands, all Palearctic *marinus *have clade 1 mtDNA haplotypes, that are also observed among the majority (21 out of 32) Nearctic *marinus *samples. This confirms that *marinus *most likely developed originally in Europe as a member of mtDNA clade 1, and only very recently moved towards the west and colonized eastern North American coasts. This process is supported by our AFLP results. STRUCTURE (Figure 4) and LAPEA (Additional File 5) analysis clearly showed that 15 Nearctic *marinus *carry autosomal signals that are identical to all 32 Palearctic *marinus *(the pale green, blue and orange genetic components). The remaining 17 Nearctic *marinus *display a unique (pale red) genetic component that is nearly fixed in birds from Lake Ontario (Figure 4). For this unique genetic component there are two possible explanations. One, it could be due to inbreeding/or a population bottleneck, both leading to a much reduced genetic heterogeneity. It is known that such individuals can group together when analyzed by STRUCTURE, and this seems to be confirmed by their reduced unbiased heterogeneity (0.23, compared to 0.29 among Palearctic *marinus*). Initially, we could not exclude a second explanation that this unique nearctic genetic component could be due to autosomal introgression by, very possibly *smithsonianus*. We investigated this possibility by a combined analysis of *marinus *together with *smithsonianus*. We were unable to discern a shared autosomal genetic component between these two gull taxa (results not shown). As a consequence, the combined mtDNA and AFLP results strongly suggest that after only a brief period of hybridization of *marinus *in the Nearctic with members of the Beringian clade [34], most likely with *smithsonianus*, they again rapidly became reproductively isolated. This process could have involved only a limited number of individuals and only lasted a limited number of generations. As such this is not an exceptional process. Although *L. marinus *is now fully reproductively isolated from all species it co-occurs with, in the mtDNA network it is nested among several taxa with known hybridization during colonization processes: *argentatus × hyperboreus *[35,36], *michahellis × graellsii *[37], *cachinnans × argentatus *[38], and earlier in the 20th century *argentatus × fuscus *[39]. This also supports the general view that reinforcement plays an important role in the evolution of reproductive isolation [40,41]. If this is also true in gulls, and our present data seems to support this, it could mean that *marinus*, after a period of allopatric divergence, has had a relatively long history of geographic contact with closely related taxa which facilitated the perfection of reproductive barriers through reinforcement multiple times. This may also explain why *marinus *achieved complete reproductive isolation more rapidly than other taxa in the herring gull group and, thus, why the phylogenetic age of the *marinus *lineage was previously overestimated.

### Phylogeographic history of circumpolar breeding *hyperboreus*

Among the circumpolar breeding glaucous gull (*L. hyperboreus*) we see an even more distinct clustering of mtDNA haplotypes into two subgroups. All *hyperboreus *from Nearctic breeding colonies display exclusively clade 2 mtDNA haplotypes, whereas Palearctic breeding *hyperboreus *display clade 1 mtDNA haplotypes (Figures 2 and 5). The only two Palearctic *hyperboreus *with clade 2 mtDNA sequences are individuals wintering on Faroe Islands. The glaucous gull is a member of the very large Beringian clade [34] that originated and diversified in the North Pacific/NW Arctic coasts of North America and North-East of Russia. Based on its clade 2 mtDNA haplotypes it is genetically very closely related to *smithsonianus *(see Figure 2). This tight genetic affinity between *hyperboreus *and *smithsonianus *is confirmed by means of our AFLP data. The STRUCTURE and LAPEA analyses on only these two taxa combined, based on all 230 AFLP loci, confirm the close relationship between these two taxa (Figure 5, Additional File 6). In addition, when analysing all taxa combined, for 230 AFLP loci and 43 selected AFLP loci, STRUCTURE combines these two taxa at lower levels (K = 2 - 4) in a majority of independent runs. From the mtDNA network it appears that *hyperboreus *only shares non-basal clade 1 haplotypes with *argentatus *(Figure 2, group B) that are obviously genetically more similar to each other than the widely spaced clade 2 mtDNA haplotypes observed among the Nearctic *hyperboreus *(Figure 2, group H and I). This suggests that after the most recent deglaciation event Nearctic *hyperboreus *spread along the most northern Palearctic coasts into northern Europe where they came into contact with *argentatus *birds of clade 1. The AFLP data shows that the North American *hyperboreus *populations are inseparable from Eurasian *hyperboreus*. If we combine the mtDNA results and AFLP results, the simplest explanation is a scenario where, on its way into Europe, *hyperboreus *lost their original mtDNA by complete introgression of *argentatus *mtDNA, but retained their original autosomal gene pool. A sufficiently long time of hybridization of *hyperboreus *with *argentatus *combined with continued skewed introgression resulted in the complete mitochondrial replacement by clade 1 haplotypes into the gene pool of European *hyperboreus*, that reaches as far east as Taimyr. Unfortunately we have no samples from further east in Russia. Obviously, only with those samples we can completely resolve this circumpolar expansion/introgression process.

## Conclusions

The present study focuses on the reconstruction of the phylogeographic history of three gull species: European herring gull (*L. argentatus*), glaucous gull (*L. hyperboreus*), and great black-backed gull (*L. marinus*). For *argentatus *and *hyperboreus *we observed a biphyletic distribution of mtDNA sequence haplotypes in a previous study [8], whereas the present study also demonstrated a biphyletic mtDNA haplotype distribution for *marinus*. For *argentatus *the mtDNA biphyletic distribution corresponded partly with a geographically distinct distribution among breeding colonies. AFLP loci indicate a remarkable complex autosomal pattern of genetic substructure. Among European herring gulls we detect signals of a number of distinct demographic events that could correspond with a repeated expansion out of the same ancestral refugium, later followed by a separation into a northern and southern population. Moreover, our data also suggest that there is a still ongoing process of introgression among all *argentatus *populations that is impossible to discern in more detail using AFLP.

For *hyperboreus*, the most likely scenario explaining their present biphyletic mtDNA distribution is a process whereby Nearctic *hyperboreus *invaded into northern *argentatus *refugia. After some time, upon complete replacement of their original clade 2 mtDNA haplotypes by *argentatus *derived clade 1 haplotypes, they again became more-or-less reproductively isolated (but see [10]). As a consequence of this process, Palearctic *hyperboreus *now strictly displays clade 1 mtDNA haplotypes, but still remain genetically more similar in their autosomes to their closely related Nearctic ancestors (also including *smithsonianus*).

The present data presents an even more recent expansion process of Palearctic *marinus *into the Nearctic. If our current AFLP and mtDNA data are reliable, the most likely scenario of *marinus *involves a very recent extension of their breeding range into Northern America that briefly involved some hybridisation with (most likely) *smithsonianus*, that seems only reflected in mtDNA.

We originally speculated that in addition to a more detailed mtDNA analyses, the use of AFLP markers could substantially supplement and clarify the complex process of population expansions among members of the herring gull assemblage. AFLP loci have proven to be very useful and informative [42,43]. In some cases, AFLP results strongly supported those based on mtDNA haplotypes among Greenish Warblers and Wild turkeys [44,45], although others report a much less clear result, e.g. among Crossbills [46], most likely due the latter's more recent "speciation" process, not unlike the situation among the herring gull assemblage. Our present study demonstrates that AFLP loci do provide additional support for a much more complex speciation process among the herring gull species assemblage. However, for a more detailed reconstruction of the timing and directions of the various processes of gene flow, we need massive autosomal sequence haplotype information. This, obviously, is the topic of our future analyses among the members of this fascinating species complex.

## Authors' contributions

VS, DLH, MR and PdK are equally responsible for the molecular lab work, the data analyses and manuscript composition. PdK, VS, and JZ performed most of the statistical data analyses, including running STRUCTURE, TFPGA, NETWORK, and LAPEA. AJH initiated the study, DLH and PdK supervised it. All authors read and approved the final manuscript.

## Supplementary Material

Additional file 1 ***Argentatus *variable nucleotide positions and accession numbers of mitochondrial hypervariable region 1**. This file contains a table of aligned variable nucleotide positions of mitochondrial *hypervariable region 1 *(*HVR1*) sequences and their Genbank accession numbers for 377 herring gull samples from 16 colonies sorted by colony membership. Dotted positions indicate identity to the reverence sequence. These sequences were used for counting the numbers of individuals belonging to clade 1 or clade 2 as shown in Figure 3. For each sequence we indicate its clade 1 or clade 2 allocation. Click here for file

Additional file 2 **UPGMA-tree based on mitochondrial hypervariable region 1 (HVR1)**. This file contains a UPGMA-tree based on sequences of hypervariable region 1. Calculation was done by using the average distance BLOSUM62 [21] routine in JALVIEW [22]. A total set of 377 *argentatus *from 16 different European colonies, 32 *marinus *from five European colonies and 32 *marinus *from three eastern North American colonies, 32 *hyperboreus *from four Eurasian colonies and 35 *hyperboreus *from four North American colonies, 35 *smithsonianus *from four North American colonies, 30 *fuscus *and *graellsii*, 31 *michahellis *and *atlantis*, and 33 *cachinnans *were sequenced on HVR1 (see also Additional files 1 and 3) and used for the tree calculation. Based on this resulting tree, all samples were assigned to either clade 1 or clade 2. Rooting of the tree was done by the use of previously published [8] Western gull (*L. occidentalis*) sequences. Click here for file

Additional file 3 **Variable positions and Genbank accession numbers of mitochondrial sequences**. This file contains a table of aligned variable nucleotide positions of mitochondrial *cytochrome b *gene and *hypervariable region 1 *sequences and their Genbank accession numbers for 368 analysed large gulls. The reference sequence is *argentatus*_0127 from Russia, White Sea. Dotted positions indicate identity to the reverence sequence. Also indicated are taxon membership, geographic origin, and sample ID for each individual. These sequences were used to calculate the median-joining networks in Figure 2 of this article. Click here for file

Additional file 4 **Laplacian Eigenfunction plot based on 230 AFLP loci among European herring gulls (*L. argentatus*)**. This file contains the two-dimensional plot of the two first Eigenvectors (and the percentage of the total variance they explain) of LAPEA on 109 herring gull individuals using all 230 AFLP loci. These LAPEA results independently confirmed the STRUCTURE results (see Figure 3). The four different colours in this LAPEA plot correspond with those in Figure 3. The first Eigenvector clearly separates the two ancestral populations indicated with blue and red from those indicated in green and yellow. The second Eigenvector provides a strong contrast between blue and red, and weakly differentiates green from yellow. Click here for file

Additional file 5 **Laplacian Eigenfunction plot based on 230 AFLP loci among great black-backed gulls (*L. marinus*)**. This file contains the two-dimensional plot of the two first Eigenvectors (and the percentage of the total variance they explain) of LAPEA on 32 Palaearctic and 32 Nearctic *marinus*. Also in *marinus *LAPEA results correspond with those from STRUCTURE (see figure 4). The four different colours in this LAPEA plot correspond with those in Figure 4. The first Eigenvector clearly separates individuals carrying contributions from the ancestral population indicated by pale orange from those carrying contributions from the ancestral population indicated with pale green. The first Eigenvector also separates (although less strongly), the individuals carrying contributions from the two less distinct ancestral populations (pale red and pale blue). The second Eigenvector separates individuals carrying contributions from the two ancestral populations indicated by pale orange and pale green from those carrying contributions from the other two ancestral populations (indicated with pale red and pale blue). Click here for file

Additional file 6 **Laplacian eigenfunction plot based on 230 AFLP loci among glaucous gulls (*L. hyperboreus*) and North American herring gulls (*L. smithsonianus*)**. This file contains the two-dimensional plot of the two first Eigenvectors (and the percentage of the total variance they explain) of LAPEA on 32 Palaearctic *hyperboreus*, 35 Nearctic *hyperboreus*, and 35 Nearctic *smithsonianus*. These LAPEA results correspond with those from STRUCTURE (see figure 5). The three different colours in this LAPEA plot correspond with those in Figure 5. The first Eigenvector clearly separates individuals carrying contributions from the ancestral population indicated by pale yellow from those carrying contributions from the ancestral populations indicated with red and pale blue. The The second Eigenvector separates individuals carrying contributions from the two ancestral populations indicated by pale yellow and red from those carrying contributions from the ancestral population indicated with pale blue. Click here for file

## References

[B1] Dobzhansky T Genetics and the origin of species 1937 New York: Columbia Univ Press

[B2] Slatkin M Gene flow and the geographic structure of natural populations Science 1987 236 787 792 10.1126/science.3576198 3576198

[B3] Turelli M Barton NH Coyne JA Theory and speciation Trends Ecol Evol 2001 16 330 343 10.1016/S0169-5347(01)02177-2 11403865

[B4] Mayr E Systematics and the origin of species 1942 New York: Columbia Univ Press

[B5] Cain AJ Animal species and their evolution 1954 London: Hutchinson House

[B6] Irwin DE Irwin HJ Price TD Ring species as bridges between microevolution and speciation Genetica 2001 112-113 223 243 10.1023/A:1013319217703 11838767

[B7] Haffer J Glutz von Blotzheim UN, Bauer KM Systematik und Taxonomie der *Larus argentatus *- Artengruppe Handbuch der Vögel Mitteleuropas 1982 8 Wiesbaden Akad Verlagsges 502 514

[B8] Liebers D de Knijff P Helbig AJ The herring hull (*Larus argentatus*) complex is not a ring species Proc Roy Soc London B, Biol Sci 2004 271 893 901 10.1098/rspb.2004.2679 PMC1691675 15255043

[B9] Geyr von Schweppenburg H Zur Systematik der *fuscus-argentatus*-Möwen J Ornithol 1938 86 345 365 10.1007/BF01947433

[B10] Vigfussdottir F Palsson S Ingolfsson A Hybridization of glaucous gull (*Larus hyperboreus*) and herring gull (*Larus argentatus*) in Iceland: mitochondrial and microsatellite data Phil Trans R Soc B 2008 363 2851 2860 10.1098/rstb.2008.0042 18508755 PMC2606735

[B11] Vos P Hogers R Bleeker M Reijans M van de Lee T Hornes M Frijters A Pot J Peleman J Kuiper M AFLP: a new technique for DNA fingerprinting Nucleic Acids Res 1995 23 4407 4414 10.1093/nar/23.21.4407 7501463 PMC307397

[B12] Barluenga M Stölting KN Salzburger W Muschick M Meyer A Sympatric speciation in Nicaraguan crater lake cichlid fish Nature 2006 439 719 723 10.1038/nature04325 16467837

[B13] Belaj A Satovic Z Cipriani G Baldoni L Testolin R Rallo L Trujillo I Comparative study of the discriminating capacity of RAPD, AFLP and SSR markers and of their effectiveness in establishing genetic relationships in olive Theor Appl Genet 2003 107 736 744 10.1007/s00122-003-1301-5 12819908

[B14] Woodhead M Russell J Squirrell J Hollingsworth PM Mackenzie K Gibby M Powell W Comparative analysis of population genetic structure in *Athyrium distentifolium *(Pteridophyta) using AFLPs and SSRs from anonymous and transcribed gene regions Mol Ecol 2005 14 1681 1695 10.1111/j.1365-294X.2005.02543.x 15836642

[B15] Sonstebo JH Borgstrom R Heun M A comparison of AFLPs and microsatellites to identify the population structure of brown trout (*Salmo trutta *L.) populations from Hardangervidda, Norway Mol Ecol 2007 16 1427 1438 10.1111/j.1365-294X.2007.03256.x 17391267

[B16] Despre's L Gielly L Redoutet B Taberlet P Using AFLP to resolve phylogenetic relationships in a morphologically diversified plant species complex when nuclear and chloroplast sequences fail to reveal variability Mol Phylogenet Evol 2003 27 185 196 10.1016/S1055-7903(02)00445-1 12695084

[B17] Kardolus JP van Eck HJ van den Berg RG The potential of AFLPs in biosystematics: a first application in Solanum taxonomy (Solanaceae) Plant Syst Evol 1998 210 87 103 10.1007/BF00984729

[B18] Perrie LR Brownsey PJ Lockhart PJ Large MF Evidence for an allopolyploid complex in New Zealand Polystichum (Dryopteridaceae) NZ J Bot 2003 41 189 215

[B19] Campbell D Duchesne P Bernatchez L AFLP utility for population assignment studies: analytical investigation and empirical comparison with microsatellites Mol Ecol 2003 12 1979 1991 10.1046/j.1365-294X.2003.01856.x 12803646

[B20] Bensch S Helbig AJ Salomon M Seibold I Amplified fragment length polymorphism analysis identifies hybrids between two subspecies of warblers Mol Ecol 2002 11 473 481 10.1046/j.0962-1083.2001.01455.x 11918782

[B21] Henikoff S Henikoff JG Amino acid substitution matrices from protein blocks PNAS 1992 89 10915 10919 10.1073/pnas.89.22.10915 1438297 PMC50453

[B22] Waterhouse AM Procter JB Martin DMA Clamp M Barton GJ Jalview Version 2 - a multiple sequence alignment editor and analysis workbench Bioinformatics 2009 25 1189 1191 10.1093/bioinformatics/btp033 19151095 PMC2672624

[B23] Bandelt HJ Forster P Röhl A Median-joining networks for inferring intraspecific phylogenies Mol Biol Evol 1999 16 37 48 10331250 10.1093/oxfordjournals.molbev.a026036

[B24] Miller MP Tools for population genetic analyses (TFPGA). A Windows^© ^program for the analysis of allozyme and molecular population genetic data. Version 1.3 Distributed by the author 1997 Department of Biological Sciences, Northern Arizona University

[B25] Falush D Stephens M Pritchard JK Inference of population structure using multilocus genotype data: dominant markers and null alleles Molecular Ecology Notes 2007 7 474 478 10.1111/j.1471-8286.2007.01758.x PMC1974779 18784791

[B26] Rosenberg NA Burke T Elo K Feldman MW Freidlin PJ Groenen MA Hillel J Maki-Tanila A Tixier-Boichard M Vignal A Wimmers K Weigend S Empirical evaluation of genetic clustering methods using multilocus genotypes from 20 chicken breeds Genetics 2001 159 699 713 11606545 10.1093/genetics/159.2.699 PMC1461842

[B27] Earl DA Structure Harvester v0.3 2009 http://users.soe.ucsc.edu/~dearl/software/struct_harvest/

[B28] Evanno G Regnaut S Goudet S Detecting the number of clusters of individuals using the software STRUCTURE: a simulation study Molecular Ecology 2005 14 2611 2620 10.1111/j.1365-294X.2005.02553.x 15969739

[B29] Jakobsson M Rosenberg NA CLUMPP: a cluster matching and permutation program for dealing with label switching and multimodality in analysis of population structure Bioinformatics 2007 23 1801 1806 10.1093/bioinformatics/btm233 17485429

[B30] Rosenberg NA DISTRUCT: a program for the graphical display of population structure Mol Ecol Notes 2004 4 137 138 10.1046/j.1471-8286.2003.00566.x

[B31] Zhang J Niyogi P McPeek MS Laplacian Eigenfunctions learn population structure PLoS ONE 2009 4 e7928 10.1371/journal.pone.0007928 19956572 PMC2779848

[B32] Jakobsson M Scholz SW Scheet P Gibbs JR VanLiere JM Fung H-C Szpiech ZA Degnan JH Wang K Guerreiro R Bras JM Schymick JC Hernandez DG Traynor BJ Simon-Sanchez J Matarin M Britton A van de Leemput J Rafferty I Bucan M Cann HM Hardy JM Rosenberg NA Singleton AB Genotype, haplotype and copy-number variation in worldwide human populations Nature 2008 451 998 1003 10.1038/nature06742 18288195

[B33] Mierauskas P Greimas E Buzun V A comparison of morphometrics, wing tip pattern and vocalizations between yellow-legged herring gulls (*Larus argentatus*) from Eastern Baltic and *Larus cachinnans* Acta Ornithol Lithuanica 1991 4 3 26

[B34] de Knijff P Helbig AJ Liebers D The Beringian connection: speciation in the herring gull assemblage of North America Am Birding 2005 37 402 411

[B35] Ingolfsson A Hybridization of glaucous gulls *Larus hyperboreus *and herring gulls *L. argentatus *in Iceland Ibis 1970 112 340 362 10.1111/j.1474-919X.1970.tb00112.x PMC2606735 18508755

[B36] Spear LB Hybridization of glaucous and herring gulls at the Mackenzie Delta, Canada Auk 1987 104 123 125

[B37] van Swelm ND Status of yellow-legged gull *Larus michahellis *as a breeding bird in the Netherlands Sula 1998 12 199 202

[B38] Panov EN Monzikov DG Intergradation between the herring gull *Larus argentatus *and the southern herring gull *Larus cachinnans *in European Russia Russian J Zool 1999 78 334 348

[B39] Tinbergen N The Herring Gull's World 1953 London: Collins

[B40] Coyne JA Orr HA "Patterns of speciation in Drosophila" revisited Evolution 1997 51 295 303 10.2307/2410984 28568795

[B41] Servedio MR Saetre GP Speciation as a positive feedback loop between postzygotic and prezygotic barriers to gene flow Proc R Soc London B 2003 270 1473 1479 10.1098/rspb.2003.2391 PMC1691397 12965012

[B42] Bensch S Akesson M Ten years of AFLP in ecology and evolution: why so few animals? Mol Ecol 2005 14 2899 2914 10.1111/j.1365-294X.2005.02655.x 16101761

[B43] Meudt HM Clarke AC Almost forgotten or latest practice? AFLP applications, analyses and advances Trends in Plant Science 2007 12 106 117 10.1016/j.tplants.2007.02.001 17303467

[B44] Irwin DE Bensch S Irwin HJ Price TD Speciation by distance in a ring species Science 2005 307 414 416 10.1126/science.1105201 15662011

[B45] Mock KE Theimer TC Rhodes OE Jr Greenberg DL Keim P Genetic variation across the historical range of the wild turkey (*Meleagris gallopavo*) Mol Ecol 2002 11 643 657 10.1046/j.1365-294X.2002.01467.x 11972754

[B46] Parchman TL Benkman CW Britch SC Patterns of genetic variation in the adaptive radiation of New World crossbills (Aves: Loxia) Mol Ecol 2006 15 1873 1887 10.1111/j.1365-294X.2006.02895.x 16689904

