# Supplementary material for: Introgressive hybridization and the evolutionary history of the herring gull complex revealed by mitochondrial and nuclear DNA
Source: BMC Evol Biol. 2010 Nov 11;10:348. doi: 10.1186/1471-2148-10-348 (PMC2993719; doi:10.1186/1471-2148-10-348)
Supplement: Additional file 1 — Argentatus variable nucleotide positions and accession numbers of mitochondrial hypervariable region 1. This file contains a table of aligned variable nucleotide positions of mitochondrial hypervariable region 1 (HVR1) sequences and their Genbank accession numbers for 377 herring gull samples from 16 colonies sorted by colony membership. Dotted positions indicate identity to the reverence sequence. These sequences were used for counting the numbers of individuals belonging to clade 1 or clade 2 as shown in Figure 3. For each sequence we indicate its clade 1 or clade 2 allocation. [file 1471-2148-10-348-S1.PDF]

Additional file 1 – *Argentatus* variable nucleotide positions and accession numbers of mitochondrial *hypervariable region 1* (*HVR1*).

```
Sample ID            mtDNA HVR1  (39 variable sites)          clade     acc. number
             0000000000 0001111111 1111111222 222222233
             1223367778 8890001112 2334588122 344899979
             1476740132 5891272681 5134113825 636735672

Reference    tttagcccgt tttaatttcg cctggtagtc gtcacgaga
```

***argentatus* (EST): Estonia, Matsalu (n=25)**

```
#argEST_1207 ...ga...ac ..c.g....a -........t .......ag clade 1  AJ508310
#argEST_1208 .a.....tac ..c.g...t. -......... ....t.g.. clade 1  AJ276950
#argEST_1209 .......... .......... .......... ..t...... clade 2  AJ276946
#argEST_1210 .......... c......... .t.......t ......... clade 2  AJ507815
#argEST_1211 .a.....tac ..c.g...t. -......... ....t.g.. clade 1  AJ276950
#argEST_1212 .a.....tac ..c.g...t. -......... ....t.g.. clade 1  AJ276950
#argEST_1213 .......... .......... .......... ......... clade 2  AJ277127
#argEST_1214 .......... ..c....... .t........ ......... clade 2  AJ507814
#argEST_1215 .......... .c........ ......c... -c....... clade 2  AJ507746
#argEST_1216 .......... .......... .......... ..t...... clade 2  AJ276946
#argEST_1217 ...ga..... .......... .......a.. ......... clade 2  AJ276947
#argEST_1218 .......... .......... .t........ ......... clade 2  AJ277128
#argEST_1219 .a.....tac ..c.g..ct. a......... ....t.g.. clade 1  FM872451
#argEST_1220 ...ga..... .......... .......a.. ......... clade 2  AJ276947
#argEST_1221 .......... .......... .......... ......... clade 2  AJ277127
#argEST_1222 .......... .......... .......... ......... clade 2  AJ277127
#argEST_1223 .a.....tac ..c.g...t. -......... ....t.g.. clade 1  AJ276950
#argEST_1224 .......... .c........ ......c... -c....... clade 2  AJ507746
#argEST_1225 .......... ..c....... .t........ ......... clade 2  AJ507814
#argEST_1226 .......... .......... .t........ ......... clade 2  AJ277128
#argEST_1227 .......... .......... .......... ..t...... clade 2  AJ276946
#argEST_1228 .......... .......... .......... a.t...... clade 2  FM872429
#argEST_1229 ....a...ac ..c.g....a -........t .......ag clade 1  FM872442
#argEST_1230 .......... c......... .t........ ......... clade 2  FM872431
#argEST_1231 .......... .......... .......... ..t...... clade 2  AJ276946
```

***argenteus* (FAR): Denmark, Faroe Islands (n=11)**

```
#argFAR_0045 .a.....tac ..c.g...t. a......... ....t.g.. clade 1  AJ276950
#argFAR_1014 .a.....tac ..c.g...t. a........t ....t.g.. clade 1  AJ508312
#argFAR_1015 .a.....tac ..c.g...t. -......... ....t.g.. clade 1  AJ276950
#argFAR_1016 .a.....tac ..c.g...t. a......... ....t.g.. clade 1  AJ276950
#argFAR_1018 .a.....tac ..c.g...t. a......... ....t.g.. clade 1  AJ276950
#argFAR_1019 .a.....tac ..c.g...t. -......... ....t.g.. clade 1  AJ276950
#argFAR_2035 .........c .......... .......... ......... clade 2  AJ508344
#argFAR_2036 ........ac ..c.gc...a -......... ......g.. clade 1  AJ508308
#argFAR_2037 .........c .......... .......... ......... clade 2  AJ508344
#argFAR_2038 .a.....tac ..c.g...t. a........t ....t.g.. clade 1  AJ508312
#argFAR_2039 .a.....tac ..c.g...t. a........t ....t.g.. clade 1  AJ508312
```

***argentatus* (FIN): Finland, Lake Saimaa (n=33)**

```
#argFIN_0146 .......... c......... .t........ ......... clade 2  FM872431
#argFIN_0147 ...ga..... .......... .......a.. ....t.... clade 2  FM872444
#argFIN_1333 .......... .......... .......... ......... clade 2  AJ277127
#argFIN_1334 .......... .......... .......... ..t...... clade 2  AJ276946
#argFIN_1335 .......... .......... .t........ ......... clade 2  AJ277128
#argFIN_1336 .a.....tac ..c.g...t. -......... ...ct.g.. clade 1   FM872450
#argFIN_1337 .......... .......... .......... ......... clade 2  AJ277127
#argFIN_1338 .......... .......... .......... ......... clade 2  AJ277127
#argFIN_1339 .......... .......... .t........ ......... clade 2  AJ277128
#argFIN_1340 .......... .......... .t........ ......... clade 2  AJ277128
#argFIN_1341 ....a...ac ..c.g....a -........t ......gag clade 1   FM872443
#argFIN_1342 .......... .......... .......... ......... clade 2  AJ277127
#argFIN_1343 .a.....tac ..c.g...t. -......... ....t.g.. clade 1   AJ276950
#argFIN_1344 .a.....tac ..c.g...t. -......... ....t.g.. clade 1   AJ276950
#argFIN_1345 ...ga..... .......... .......a.. ......... clade 2  AJ276947
#argFIN_1346 .a.....tac ..c.g...t. -......... ....t.g.. clade 1   AJ276950
#argFIN_1347 .......... .......... .......... ..t...... clade 2  AJ276946
#argFIN_1348 .......... .......... .......... ......... clade 2  AJ277127
#argFIN_1350 .......... .......... .t........ ......... clade 2  AJ277128
#argFIN_1351 ...ga..... .......... .......a.. ......... clade 2  AJ276947
#argFIN_1352 .......... .......... .......... ......... clade 2  AJ277127
#argFIN_1471 ...ga..... .......... .......a.. ......... clade 2  AJ276947
```

#argFIN_1472 .... clade 2 AJ277127
#argFIN_1473 .... clade 2 AJ277127
#argFIN_1474 ...ga..... ....a.. .... clade 2 AJ276947
#argFIN_1475 .a.....tac ..c.g...t. -......... ....t.g.. clade 1 AJ276950
#argFIN_2166 .a.....tac ..c.g...t. -......... ....t.g.. clade 1 AJ276950
#argFIN_2167 .... .t........ .... clade 2 AJ277128
#argFIN_2168 .... .t........ .... clade 2 AJ277128
#argFIN_2171 ...ga...ac ..c.g....a -........t ....ag clade 1 AJ508310
#argFIN_2172 .a.g...tac ..c.g...t. -......... ......g.. clade 1 AJ508325
#argFIN_2173 .... clade 2 AJ277127
#argFIN_2174 ...ga..... ....a.. .... clade 2 AJ276947

***argenteus* (FRA): France, Finistere (n=27)**
#argFRA_2040 .... clade 2 AJ277127
#argFRA_2041 ....c....... .... clade 2 AJ508345
#argFRA_2042 ....ac ..c.g....a ....g.. clade 1 AJ508307
#argFRA_2043 .... clade 2 AJ277127
#argFRA_2044 ....a...ac ..c.....t. a......... ..t..at.. clade 1 AJ277133
#argFRA_2045 .... clade 2 AJ277127
#argFRA_2046 .... clade 2 AJ277127
#argFRA_2047 ......t... .... clade 2 FM872438
#argFRA_2048 .... clade 2 AJ277127
#argFRA_2049 .... clade 2 AJ277127
#argFRA_2050 ...ga...ac ..c.....t. -......... ..t..at.. clade 1 FM872446
#argFRA_2051 .... clade 2 AJ277127
#argFRA_2052 .... clade 2 AJ277127
#argFRA_2053 .... clade 2 AJ277127
#argFRA_2054 ....c .... clade 2 AJ508344
#argFRA_2055 ....ac ..c.g....a -......... ......g.. clade 1 AJ508307
#argFRA_2056 ....c .... clade 2 AJ508344
#argFRA_2057 ...ga..... .... .t.....a.. .... clade 2 AJ507809
#argFRA_2058 .... clade 2 AJ277127
#argFRA_2059 ....a...ac ..c.....t. a......... ..t..at.. clade 1 AJ277133
#argFRA_2060 ....g.. clade 2 AJ507737
#argFRA_2061 ....c....... .... clade 2 AJ508345
#argFRA_2062 ....a..... ....t...... clade 2 AJ507808
#argFRA_2063 ....a. .... clade 2 AJ508346
#argFRA_2064 .... clade 2 AJ277127
#argFRA_2065 .... clade 2 AJ277127
#argFRA_2066 .... clade 2 AJ277127

***argenteus* (HGL): Germany, Helgoland (n=40)**
#ateHGL_1392 ...ga..... ....a.. .... clade 2 AJ276947
#ateHGL_1393 ....t...... clade 2 AJ276946
#ateHGL_1394 ....tac ....g..... -......... ......g.. clade 1 FM866311
#ateHGL_1395 .a.....tac ..c.g...t. -......... ....t.g.. clade 1 AJ276950
#ateHGL_1396 .... clade 2 AJ277127
#ateHGL_1397 .... clade 2 AJ277127
#ateHGL_1398 ...ga..... ....a.. .... clade 2 AJ276947
#ateHGL_1400 .... clade 2 AJ277127
#ateHGL_1401 ....t...... clade 2 AJ276946
#ateHGL_1402 .... .t........ .... clade 2 AJ277128
#ateHGL_1403 ....t...... clade 2 AJ276946
#ateHGL_1414 ....t...... clade 2 AJ276946
#ateHGL_1415 .... clade 2 AJ277127
#ateHGL_1416 ....ac ....g....a -......... ......g.. clade 1 FM866312
#ateHGL_1417 ....t...... clade 2 AJ276946
#ateHGL_1418 ....t...... clade 2 AJ276946
#ateHGL_1419 ....t...... clade 2 AJ276946
#ateHGL_1420 .... clade 2 AJ277127
#ateHGL_1421 .... clade 2 AJ277127
#ateHGL_1423 ....t...... clade 2 AJ276946
#argHGL_2622 ....t...... clade 2 AJ276946
#argHGL_2623 ....t...... clade 2 AJ276946
#argHGL_2624 ....t...... clade 2 AJ276946
#argHGL_2625 .... clade 2 AJ277127
#argHGL_2626 ....t...... clade 2 AJ276946
#argHGL_2627 ....t...... clade 2 AJ276946
#argHGL_2628 ....t...... clade 2 AJ276946
#argHGL_2629 ....t...... clade 2 AJ276946
#argHGL_2630 ...ga..... ....a.. ..t...... clade 2 FM872445
#argHGL_2631 ...ga..... ....a.. .... clade 2 AJ276947
#argHGL_2632 ...ga..... ....a.. .... clade 2 AJ276947
#argHGL_2633 ....gg..... ....t...... clade 2 FM872430
#argHGL_2634 ....t...... clade 2 AJ276946
#argHGL_2635 ....t...... clade 2 AJ276946

```
#argHGL_2636 .......... .......... .......... ..t...... clade 2  AJ276946
#argHGL_2637 ...ga...ac ..c.g....a -........t .......ag clade 1  AJ508310
#argHGL_2638 ........ac ....g....a -......... ....t.g.. clade 1  FM872435
#argHGL_2639 .......... .......... .......... ..t...... clade 2  AJ276946
#argHGL_2640 .a.....tac ..c.g...t. a......... ....t.g.. clade 1  AJ276950
#argHGL_2641 .......... .......... .......a.. ......... clade 2  FM866319
```

***argentatus* (SSW): Schweden, NW Skane (n=20)**

```
#argSSW_2577 .......... .......... .......a.. ......... clade 2  FM866319
#argSSW_2578 .a.....tac ..c.g...t. -......... ....t.g.. clade 1  AJ276950
#argSSW_2579 ...ga..... .......... .......a.. ......... clade 2  AJ276947
#argSSW_2580 .......... .......... .......... ..t...... clade 2  AJ276946
#argSSW_2581 .a.....tac ..c.g...t. .......... ....t.g.. clade 1  AJ276950
#argSSW_2582 .......... .......... .......... ..t...... clade 2  AJ276946
#argSSW_2583 .......... .......... .......... ......... clade 2  AJ277127
#argSSW_2584 .a.....tac ..c.g...t. a......... ....t.g.. clade 1  AJ276950
#argSSW_2585 .a.....tac ..c.g...t. a......... ....t.g.. clade 1  AJ276950
#argSSW_2586 .......... .......... .......... ......... clade 2  AJ277127
#argSSW_2587 .......... .......... .......a.. ......... clade 2  FM866319
#argSSW_2588 .a.....tac .cc.g...t. -......... ....t.g.. clade 1  FM872455
#argSSW_2589 .......... .......... .......... ......... clade 2  AJ277127
#argSSW_2590 .......... .......... .......a.. ......... clade 2  FM866319
#argSSW_2591 .......... .......... .......... ......... clade 2  AJ277127
#argSSW_2592 .......... .......... .......... ..t...... clade 2  AJ276946
#argSSW_2593 .......... .......... .......a.. ......... clade 2  FM866319
#argSSW_2594 .......... .......... .......... ......... clade 2  AJ277127
#argSSW_2595 .......... ..c....... .t........ ......... clade 2  AJ507814
#argSSW_2596 .......... .......... .......... ......... clade 2  AJ277127
```

***argentatus* (MVP): Germany, Hiddensee (n=34)**

```
#argMVP_0026 .......... .......... .......... ..t...... clade 2  AJ276946
#argMVP_0027 .......... .......... .......... ......... clade 2  AJ277127
#argMVP_0028 .......... .......... .......... ..t...... clade 2  AJ276946
#argMVP_0029 .......... .......... .......... ......... clade 2  AJ277127
#argMVP_0030 .......... .......... .......... ..t...... clade 2  AJ276946
#argMVP_0031 .......... .......... .......... ..t...... clade 2  AJ276946
#argMVP_0032 .......... ..c....... .......... ......... clade 2  AJ508345
#argMVP_0902 .a.g...tac ..c.g...t. -......... ....t.g.. clade 1  AJ508315
#argMVP_0903 .......... .......... .......... ......... clade 2  AJ277127
#argMVP_0904 .......... .......... .......... ......... clade 2  AJ277127
#argMVP_0905 .......... .......... .......... ..t...... clade 2  AJ276946
#argMVP_0906 .......... .......... .......... ......... clade 2  AJ277127
#argMVP_0907 .a.....tac ..c.g...t. a......... ....t.g.. clade 1  AJ276950
#argMVP_0909 .......... .......... .......... ......... clade 2  AJ277127
#argMVP_0910 .......... .......... .......... ..t...... clade 2  AJ276946
#argMVP_0911 .......... .......... .......... ......... clade 2  AJ277127
#argMVP_2602 .......... .......... .......... ..t...... clade 2  AJ276946
#argMVP_2603 .......... .......... .t........ ......... clade 2  AJ277128
#argMVP_2604 .......... .......... .......... ..t...... clade 2  AJ276946
#argMVP_2605 .......... .......... .......a.. ......... clade 2  FM866319
#argMVP_2606 .a.....tac ..c.g...t. a......... ....t.g.. clade 1  AJ276950
#argMVP_2607 .......... .......... .......... ......... clade 2  AJ277127
#argMVP_2608 .......... .......... .t........ ......... clade 2  AJ277128
#argMVP_2609 .......... .......... .t........ ......... clade 2  AJ277128
#argMVP_2610 .......... .......... .......a.. ......... clade 2  FM866319
#argMVP_2612 .......... .......... .......... ......... clade 2  AJ277127
#argMVP_2613 .......... .......... .......... ......... clade 2  AJ277127
#argMVP_2614 .a.....tac ..c.g...t. a..a...... ....t.g.. clade 1  AJ508313
#argMVP_2615 .a.....tac ..c.g...t. -......... ....t.g.. clade 1  AJ276950
#argMVP_2616 .......... .......... .......... ..t...... clade 2  AJ276946
#argMVP_2617 .......... .......... .......... ......... clade 2  AJ277127
#argMVP_2618 .......... ..c....... .t........ ......... clade 2  AJ507814
#argMVP_2619 .......... .......... .......... ..t...... clade 2  AJ276946
#argMVP_2621 .......... .......... .......... ......... clade 2  AJ277127
```

***argentatus* (DEN): Denmark, Lindholm (n=21)**

```
#argDAN_2506 .......... .......... .......a.. ......... clade 2  FM866319
#argDAN_2507 .......... .......... .......... ......... clade 2  AJ277127
#argDAN_2508 .......... .......... .......... ......... clade 2  AJ277127
#argDAN_2509 .......... .......... .......... ......... clade 2  AJ277127
#argDAN_2510 .......... .......... .......... ..t...... clade 2  AJ276946
#argDAN_2511 ...ga..... .......... .......a.. ......... clade 2  AJ276947
#argDAN_2513 .a.....tac .cc.g...t. -......... ....t.g.. clade 1  FM872455
#argDAN_2514 .......... .......... .......... ......... clade 2  AJ277127
#argDAN_2519 .a.....tac ..c.g...t. -......... ....t.g.. clade 1  AJ276950
#argDAN_2520 ........ac ....g....a -......... ......... clade 1  FM872433
```

```
#argDAN_2521 ...ga..... .......... .......a.. ......... clade 2  AJ276947
#argDAN_2541 .a.....tac ..c.g...t. -......... ....t.g.. clade 1  AJ276950
#argDAN_2542 .a.....tac .cc.g...t. -......... ....t.g.. clade 1  FM872455
#argDAN_2543 .a.....tac .cc.g...t. -......... ....t.g.. clade 1  FM872455
#argDAN_2544 ...ga..... .......... .......a.. ......... clade 2  AJ276947
#argDAN_2545 ...ga..... .......... .......a.. ......... clade 2  AJ276947
#argDAN_2546 .......... .......... .......... ..t...... clade 2  AJ276946
#argDAN_2547 .......... .......... .......... ..t...... clade 2  AJ276946
#argDAN_2548 .......... .......... .......... ......... clade 2  AJ277127
#argDAN_2549 .a.....tac ..c.g...t. -......... ....t.g.. clade 1  AJ276950
#argDAN_2550 .a.....tac ..c.g...t. -......... ....t.g.. clade 1  AJ276950
```

argenteus **(ENG): England, Isle of May (n=21)**

```
#ateENG_2781 ....a..... .......... .......... ......... clade 2  FM872439
#ateENG_2782 ....a..... .......... .......... ......... clade 2  FM872439
#ateENG_2783 ...ga..... .......... .......a.. ......... clade 2  AJ276947
#ateENG_2784 .......... .......... .......... ......... clade 2  AJ277127
#ateENG_2785 ...ga..... .......... .......a.. ......... clade 2  AJ276947
#ateENG_2786 ...ga..... .......... .......a.. ......... clade 2  AJ276947
#ateENG_2787 ...ga..... .......... .......a.. ......... clade 2  AJ276947
#ateENG_2788 .......... .......... .......... ......... clade 2  AJ277127
#ateENG_2789 .......... .......... .......... ......... clade 2  AJ277127
#ateENG_2790 .......... .......... .......... ......... clade 2  AJ277127
#ateENG_2823 .......... .......... .......... ......... clade 2  AJ277127
#ateENG_2824 .......... .......... .......... ......... clade 2  AJ277127
#ateENG_2826 ...ga..... .......... .......a.. ......... clade 2  AJ276947
#ateENG_2827 .........c .......... .......... ......... clade 2  AJ508344
#ateENG_2828 ........ac ..c.g....a -......... ......g.. clade 1   AJ508307
#ateENG_2829 c..ga..... .......... .......a.. ......... clade 2  FM866319
#ateENG_2830 c........c .......... .......... ......... clade 2  FM866320
#ateENG_2831 ........ac ....g....a -......... ......g.. clade 1   FM866312
#ateENG_2832 ........ac ....g....- .......... ......g.. clade 1   FM866315
#ateENG_2833 ....a...ac ..c.....t. -......... ..t..at.. clade 1   AJ277133
#ateENG_2834 ....a...a. ..c....... .......... ......g.. clade 2  FM872441
```

argenteus **(ICE): Iceland, Skruder (n=23)**

```
#ateICE_2080 .......... .......... .....c.... ......... clade 2  AJ508343
#ateICE_2081 .a.....tac ..c.g...t. a......... ....t.g.. clade 1  AJ276950
#ateICE_2082 ....a...ac ..c.....t. -......... ..t..at.. clade 1  AJ277133
#ateICE_2083 .a.....tac ..c.g...t. a........t ....t.g.. clade 1  AJ508312
#ateICE_2084 .......... .......... .......... ..t...... clade 2  AJ276946
#ateICE_2085 .a.....tac ..c.g...t. -......... ....t.g.. clade 1  AJ276950
#ateICE_2086 ........ac ..c.g....a -......... ......g.. clade 1  AJ508307
#ateICE_2087 .........c .......... .......... ......... clade 2  AJ508344
#ateICE_2088 .......... .......... .......... ..t...... clade 2  AJ276946
#ateICE_2089 .a.....tac .cc.gc..t. -...a..... ....t.... clade 1  AJ508314
#ateICE_2090 ...ga..... .......... .......a.. ......... clade 2  AJ276947
#ateICE_2091 .......... .......... ..c....... ......... clade 2  FM866314
#ateICE_2092 .a.....tac ..c.g...t. a........t ....t.g.. clade 1  AJ508312
#ateICE_2093 ...ga..... .......... .......a.. ......... clade 2  AJ276947
#ateICE_2094 ...ga..... .......... .......a.. ......... clade 2  AJ276947
#ateICE_2095 .a.....tac ..c.g...t. a......... ....t.g.. clade 1  AJ276950
#ateICE_2096 .......... .......... .......... ......... clade 2  AJ277127
#ateICE_2097 .......... .......... .......... ..t...... clade 2  AJ276946
#ateICE_2098 .a.....tac ..c.g...t. a........t ....t.g.. clade 1  AJ508312
#ateICE_2099 ........ac ....g....- .......... ......g.. clade 1  FM866315
#ateICE_2100 ........ac ....g....- .......... ......g.. clade 1  FM866315
#ateICE_2101 ...ga..... .......... .......a.. ......... clade 2  AJ276947
#ateICE_2102 .a.....tac ..c.g...t. a........t ....t.g.. clade 1  AJ508312
```

argenteus **(ICW): Iceland, Karlsskali (n=14)**

```
#ateICW_2103 .a.....tac ..c.g...t. a........t ....t.g.. clade 1  AJ508312
#ateICW_2104 ........ac ..c.gc...a -......... ......g.. clade 1  AJ508308
#ateICW_2105 .a.....tac ..c.g...t. -......... ....t.g.. clade 1  AJ276950
#ateICW_2106 .a.....tac ..c.g...t. -...a...c. ...tt.g.. clade 1  AJ508311
#ateICW_2107 ...ga..... .......... .......a.. ......... clade 2  AJ276947
#ateICW_2108 ...ga..... .......... .......a.. ......... clade 2  AJ276947
#ateICW_2109 .......... .......... .......... ..t...... clade 2  AJ276946
#ateICW_2110 .......tac ..c.g....a -......... ......g.. clade 1  FM866308
#ateICW_2111 .a.....tac ..c.g...t. a......... ....t.g.. clade 1  AJ276950
#ateICW_2112 .a.....tac ..c.g...t. -......... ....t.g.. clade 1  AJ276950
#ateICW_2113 ........ac ....g....a -......... ......g.. clade 1  FM866312
#ateICW_2114 .......... .......... .......... ..t...... clade 2  AJ276946
#ateICW_2115 ...ga..... .......... .......a.. ......... clade 2  AJ276947
#ateICW_2116 ...ga...ac ..c.gc...a -......... ......g.. clade 1  FM872447
```

***argentatus* (POL): Poland, Wloclawek (n=17)**

```
#argPOL_0189 .......... .......... .......... ......... clade 2  AJ277127
#argPOL_0190 .......... .......... .......... ......... clade 2  AJ277127
#argPOL_0191 .......... .......... .......... ......... clade 2  AJ277127
#argPOL_0192 .......... .......... .......... ..t...... clade 2  AJ276946
#argPOL_0193 .......... .......... .......... ......... clade 2  AJ277127
#argPOL_0196 .......... c......... .t........ ......... clade 2  FM872431
#argPOL_0198 .......... c......... .t........ ......... clade 2  FM872431
#argPOL_0200 ...ga..... .......... .......a.. ......... clade 2  AJ276947
#argPOL_0202 .a.....tac ..c.g...t. -......... ....t.g.. clade 1  AJ276950
#argPOL_0203 .......... .......... .......... ......... clade 2  AJ277127
#argPOL_0204 ..cg...... ....g.c... .......... .....a.ag clade 2  FM872448
#argPOL_0205 ...ga..... .......... .......a.. ......... clade 2  AJ276947
#argPOL_0206 .......... .......... .......... ..t...... clade 2  AJ276946
#argPOL_0207 ...ga..... .......... .......a.. ......... clade 2  AJ276947
#argPOL_0208 .......... .......... .t........ ......... clade 2  AJ277128
#argPOL_0209 .......... .......... .......... ......... clade 2  AJ277127
#argPOL_0210 .......... .......... .......... ..t...... clade 2  AJ276946
```

***argentatus* (NNO): Norway, Tromso (n=14)**

```
#argNNO_2117 .a.....tac ..c.g...t. -......... ....t.g.. clade 1  AJ276950
#argNNO_2118 .a.....tac ..c.g...t. -......... ....t.g.. clade 1  AJ276950
#argNNO_2119 .a.....tac ..c.g...t. -......... ....t.g.. clade 1  AJ276950
#argNNO_2120 ...ga..... .......... .......a.. ......... clade 2  AJ276947
#argNNO_2121 .a.....tac ..c.g...t. a......... ....t.g.. clade 1  AJ276950
#argNNO_2122 ...ga..... .......... .......a.. ......... clade 2  AJ276947
#argNNO_2123 .a.....tac ..c.g...t. -......... ....t.g.. clade 1  AJ276950
#argNNO_2124 .a.....tac ..c.g...t. -......... a...t.g.. clade 1  AJ276950
#argNNO_2125 .a.....tac ..c.g...t. a......... ....t.g.. clade 1  AJ276950
#argNNO_2126 .....t..ac ..c.g....a -......... ......g.. clade 1  AJ508309
#argNNO_2127 .a.....tac ..c.g...t. -......... ....t.g.. clade 1  AJ276950
#argNNO_2128 .a.....tac ..c.g...t. -......... ....t.g.. clade 1  AJ276950
#argNNO_2129 .a.....tac ..c.g...t. -......... ....t.g.. clade 1  AJ276950
#argNNO_2130 ....a..... ..c....... .......a.. ......... clade 2  FM872440
```

***argentatus* (SNO)*:* Norway, Vest-Agder (n=20)**

```
#argSNO_1431 .......... .......... .......... ......... clade 2  AJ277127
#argSNO_1432 ........ac ....g....a -......... ......g.. clade 1  FM866312
#argSNO_1433 .a.....tac .cc.g..... -......... ....t.g.. clade 1  FM872454
#argSNO_1434 .a.....tac ..c.g..ct. a......... ....t.g.. clade 1  FM872451
#argSNO_1435 .a.....tac ..c.g...t. -......... ....t.g.. clade 1  AJ276950
#argSNO_1436 .a.....tac ..c.g...t. -......... ....t.g.. clade 1  AJ276950
#argSNO_1437 .a.....tac ..c.g...ta -......... ....t.g.. clade 1  AJ276952
#argSNO_1438 .a.....tac ..c.g...t. -......... ....t.g.. clade 1  AJ276950
#argSNO_1439 .a.....tac ..c.g...t. a......... ....t.g.. clade 1  AJ276950
#argSNO_1440 .a.....tac ..c.g...t. -......... ....t.g.. clade 1  AJ276950
#argSNO_1441 .a.....tac ..c.g...t. -......... ....t.g.. clade 1  AJ276950
#argSNO_1442 .a.....tac ..c.g...t. -......... ....t.g.. clade 1  AJ276950
#argSNO_1443 .a.....tac ..c.g...t. -......... ....t.g.. clade 1  AJ276950
#argSNO_1444 .a.....tac ..c.g...t. a......... ....t.g.. clade 1  AJ276950
#argSNO_1445 .a.....tac ..c.g...t. -......... ....t.g.. clade 1  AJ276950
#argSNO_1446 .......... .......... .......... ..t...... clade 2  AJ276946
#argSNO_1447 .a.....tac ....g...t. -......... ....t.g.. clade 1  AJ508317
#argSNO_1448 .a.....tac ..c.g...t. a..a...... ....t.g.. clade 1  AJ508313
#argSNO_1449 ...ga..... .......... .......a.. ......... clade 2  AJ276947
#argSNO_1450 .......... .......... .......... ......... clade 2  AJ277127
```

***argentatus* (WSA): Russia, White Sea (n=31)**

```
#argWSE_0019 .......... .......... .t........ ......... clade 2  AJ277128
#argWSE_0022 .a.....tac ..c.g...t. -......... ....t.g.. clade 1  AJ276950
#argWSE_0023 ........ac ..c.....t. -......... ..t..at.. clade 1  FM872436
#argWSE_0024 .a.....tac ..c.g.c.t. -......... ....t.g.. clade 1  FM872452
#argWSE_0025 .a.....tac ..c.g.c.t. -......... ....t.g.. clade 1  FM872452
#argWSE_0122 .......tac ..c.g...t. -......... ....t.g.. clade 1  FM872437
#argWSE_0123 ........a. ..c.g....a -........t .......a. clade 1  FM872432
#argWSE_0124 .a.....tac ..c.g.c.t. -......... ....tag.. clade 1  FM872453
#argWSE_0125 .a.....tac ..c.g...t. -......... ....t.g.. clade 1  AJ276950
#argWSE_0126 .a.....tac ..c.g.c.t. -......... ....tag.. clade 1  FM872453
#argWSE_0127 .a.....tac ..c.g...t. -......... ....t.g.. clade 1  AJ276950
#argWSE_0128 .a.....tac ..c.g...t. -......... ....t.g.. clade 1  AJ276950
#argWSE_0129 ...ga..... .......... .......a.. ......... clade 2  AJ276947
#argWSE_0130 .a.....tac ..c.g...t. -......... ....tag.. clade 1  FM872449
#argWSE_0131 .a.....tac ..c.g...t. -......... ...ct.g.. clade 1  FM872450
#argWSE_0132 .a.....tac ..c.g...t. -......... ....t.g.. clade 1  AJ276950
#argWSE_0133 ...ga..... .......... .......a.. ......... clade 2  AJ276947
#argWSE_0134 ....a...ac ..c.g....a -........t ......gag clade 1  FM872443
```

```
#argWSE_0218 .a.....tac ..c.g...t. -......... ....t.g.. clade 1  AJ276950
#argWSE_0219 .a.....tac ..c.g...t. -......... ....t.g.. clade 1  AJ276950
#argWSE_0570 .a.ga..tac ..c.g...t. -......... ....taga. clade 1  FM872456
#argWSE_0571 .a.....tac ..c.g...t. -......... ....t.g.. clade 1  AJ276950
#argWSE_0898 .a.....tac ..c.g...ta -......... ....t.g.. clade 1  AJ276952
#argWSE_0899 .a.....tac ..c.g...ta -......... ....t.g.. clade 1  AJ276952
#argWSE_0900 .a.....tac ..c.g...t. -......... ....t.g.. clade 1  AJ276950
#argWSE_0901 .a.....tac ..c.g...ta -......... ....t.g.. clade 1  AJ276952
#argWSE_2175 .a.....tac ..c.g...t. -......... ....t.g.. clade 1  AJ276950
#argWSE_2176 .a.....tac ..c.g...t. -......... ....t.g.. clade 1  AJ276950
#argWSE_2177 .a.....tac ..c.g...t. -......... ....t.g.. clade 1  AJ276950
#argWSE_2178 .a.....tac ..c.g...t. -......... ....t.g.. clade 1  AJ276950
#argWSE_2179 .a.....tac ..c.g...t. -......... ....t.g.. clade 1  AJ276950
```

***argenteus* (NET): Netherlands, Maasvlakte (n=26)**

```
#ateNET_0778 .......... .......... .......... ......... clade 2  AJ277127
#ateNET_0779 .......... .......... .......... ......... clade 2  AJ277127
#ateNET_0780 .......... .......... .......... ......... clade 2  AJ277127
#ateNET_0781 .......... .......... .......... ......... clade 2  AJ277127
#ateNET_0782 .......... .......... .......... ..t...... clade 2  AJ276946
#ateNET_0783 .......... .......... .......... ......... clade 2  AJ277127
#ateNET_0784 .......... .......... .......... ..t...... clade 2  AJ276946
#ateNET_0785 .........c .......... .......... ......... clade 2  AJ508344
#ateNET_0786 .......... .......... .......... ......... clade 2  AJ277127
#ateNET_0787 .......... .......... .......... ..t...... clade 2  AJ276946
#ateNET_0788 .......... .......... .......a.. ......... clade 2  FM866319
#ateNET_0789 .......... .......... .......... ......... clade 2  AJ277127
#ateNET_0797 .......... .......... .......... ......... clade 2  AJ277127
#ateNET_0800 .......... .......... .......... ......... clade 2  AJ277127
#ateNET_0802 ........ac ....g....a -......... ......ga. clade 1  FM872434
#ateNET_0804 .......... .......... .......a.. ......... clade 2  FM866319
#ateNET_0805 .......... ..c....... .......... ......... clade 2  AJ508345
#ateNET_0806 .......tac ..c.g....a -......... ......g.. clade 1  FM866308
#ateNET_2180 .......... .......... .......... ......... clade 2  AJ277127
#ateNET_2182 .......... .......... .......... ......... clade 2  AJ277127
#ateNET_2183 .......... .......... .......... ......... clade 2  AJ277127
#ateNET_2184 .......... .......... .......... ..t...... clade 2  AJ276946
#ateNET_2185 .......... .......... .......... ......... clade 2  AJ277127
#ateNET_2186 .......... .......... .......... ..t...... clade 2  AJ276946
#ateNET_2188 .......... .......... .......... ......... clade 2  AJ277127
#ateNET_2189 .......... .......... .......... ......... clade 2  AJ277127
```
