# Supplementary figures and images for: Introgressive hybridization and the evolutionary history of the herring gull complex revealed by mitochondrial and nuclear DNA
Source: BMC Evol Biol. 2010 Nov 11;10:348. doi: 10.1186/1471-2148-10-348 (PMC2993719; doi:10.1186/1471-2148-10-348)

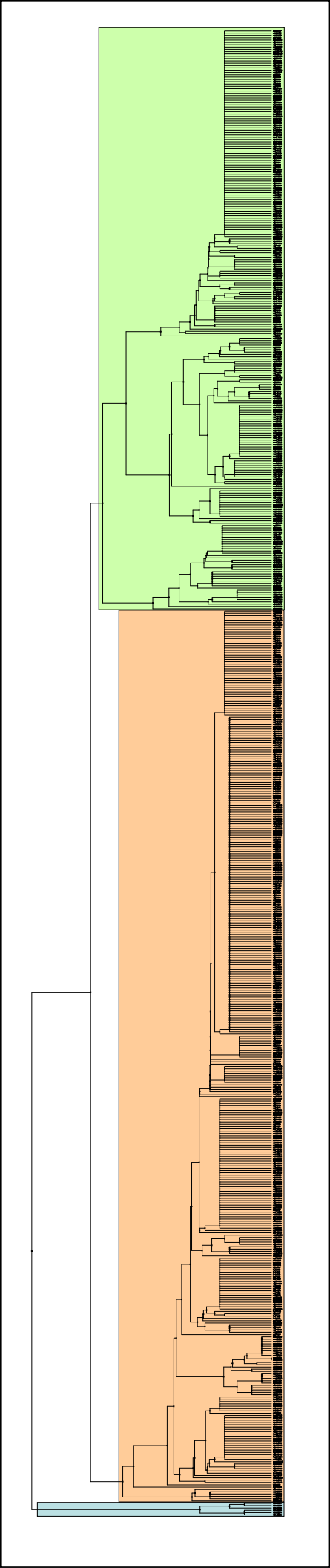

Supplement: Additional file 2 — UPGMA-tree based on mitochondrial hypervariable region 1 (HVR1). This file contains a UPGMA-tree based on sequences of hypervariable region 1. Calculation was done by using the average distance BLOSUM62 [21] routine in JALVIEW [22]. A total set of 377 argentatus from 16 different European colonies, 32 marinus from five European colonies and 32 marinus from three eastern North American colonies, 32 hyperboreus from four Eurasian colonies and 35 hyperboreus from four North American colonies, 35 smithsonianus from four North American colonies, 30 fuscus and graellsii, 31 michahellis and atlantis, and 33 cachinnans were sequenced on HVR1 (see also Additional files 1 and 3) and used for the tree calculation. Based on this resulting tree, all samples were assigned to either clade 1 or clade 2. Rooting of the tree was done by the use of previously published [8] Western gull (L. occidentalis) sequences. [file 1471-2148-10-348-S2.PDF]

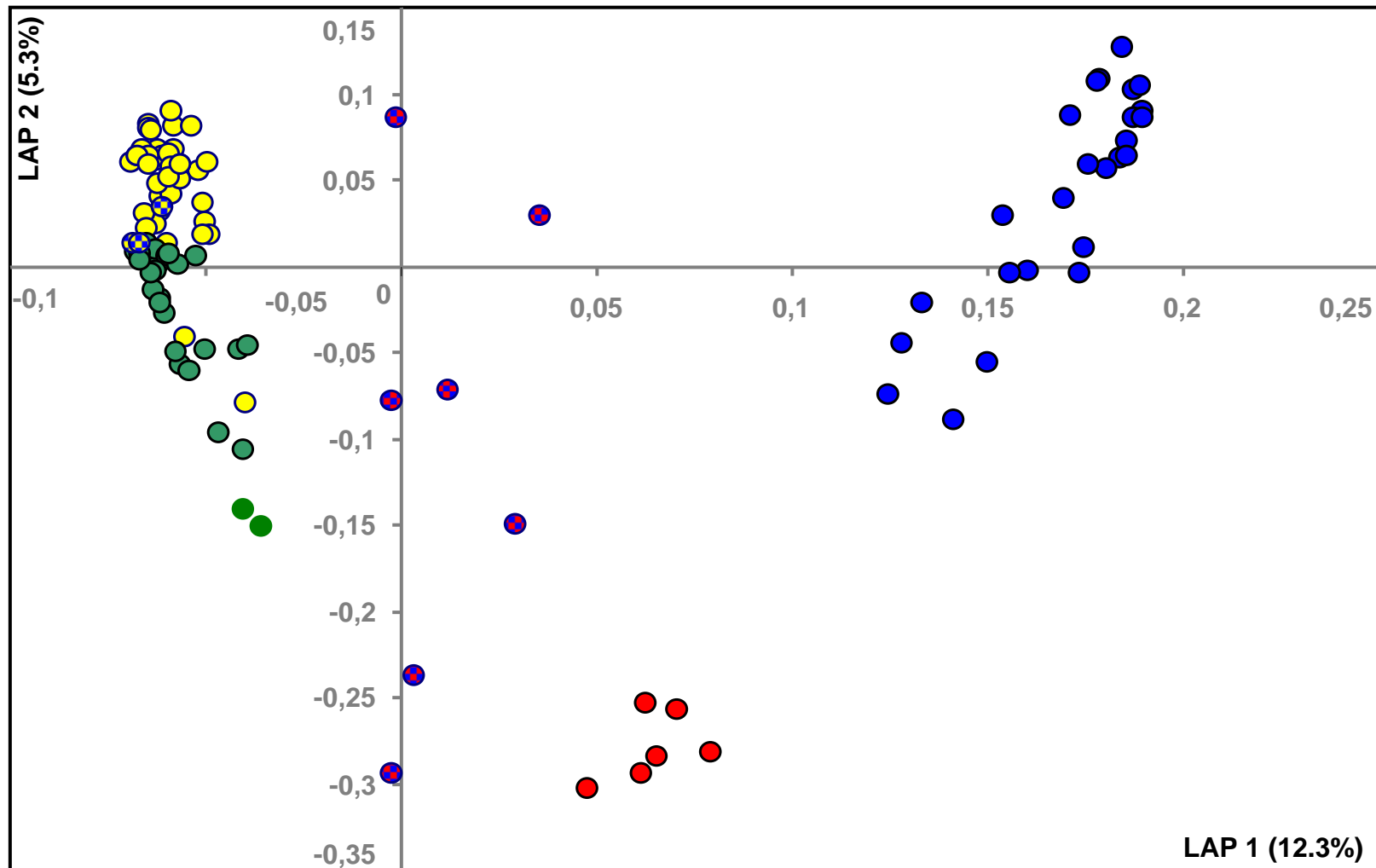

Supplement: Additional file 4 — Laplacian Eigenfunction plot based on 230 AFLP loci among European herring gulls (L. argentatus). This file contains the two-dimensional plot of the two first Eigenvectors (and the percentage of the total variance they explain) of LAPEA on 109 herring gull individuals using all 230 AFLP loci. These LAPEA results independently confirmed the STRUCTURE results (see Figure 3). The four different colours in this LAPEA plot correspond with those in Figure 3. The first Eigenvector clearly separates the two ancestral populations indicated with blue and red from those indicated in green and yellow. The second Eigenvector provides a strong contrast between blue and red, and weakly differentiates green from yellow. [file 1471-2148-10-348-S4.PDF]

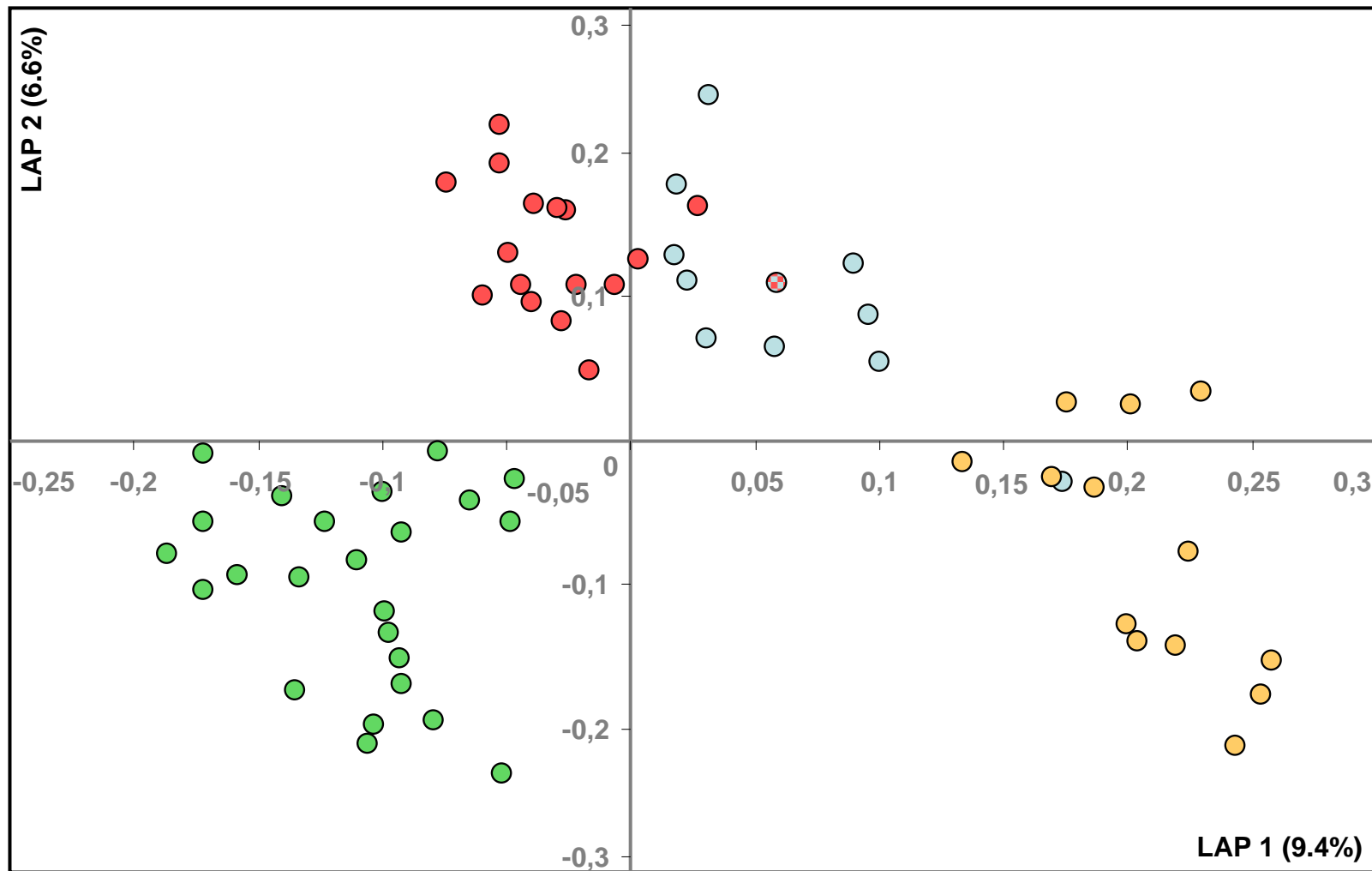

Supplement: Additional file 5 — Laplacian Eigenfunction plot based on 230 AFLP loci among great black-backed gulls (L. marinus). This file contains the two-dimensional plot of the two first Eigenvectors (and the percentage of the total variance they explain) of LAPEA on 32 Palaearctic and 32 Nearctic marinus. Also in marinus LAPEA results correspond with those from STRUCTURE (see figure 4). The four different colours in this LAPEA plot correspond with those in Figure 4. The first Eigenvector clearly separates individuals carrying contributions from the ancestral population indicated by pale orange from those carrying contributions from the ancestral population indicated with pale green. The first Eigenvector also separates (although less strongly), the individuals carrying contributions from the two less distinct ancestral populations (pale red and pale blue). The second Eigenvector separates individuals carrying contributions from the two ancestral populations indicated by pale orange and pale green from those carrying contributions from the other two ancestral populations (indicated with pale red and pale blue). [file 1471-2148-10-348-S5.PDF]

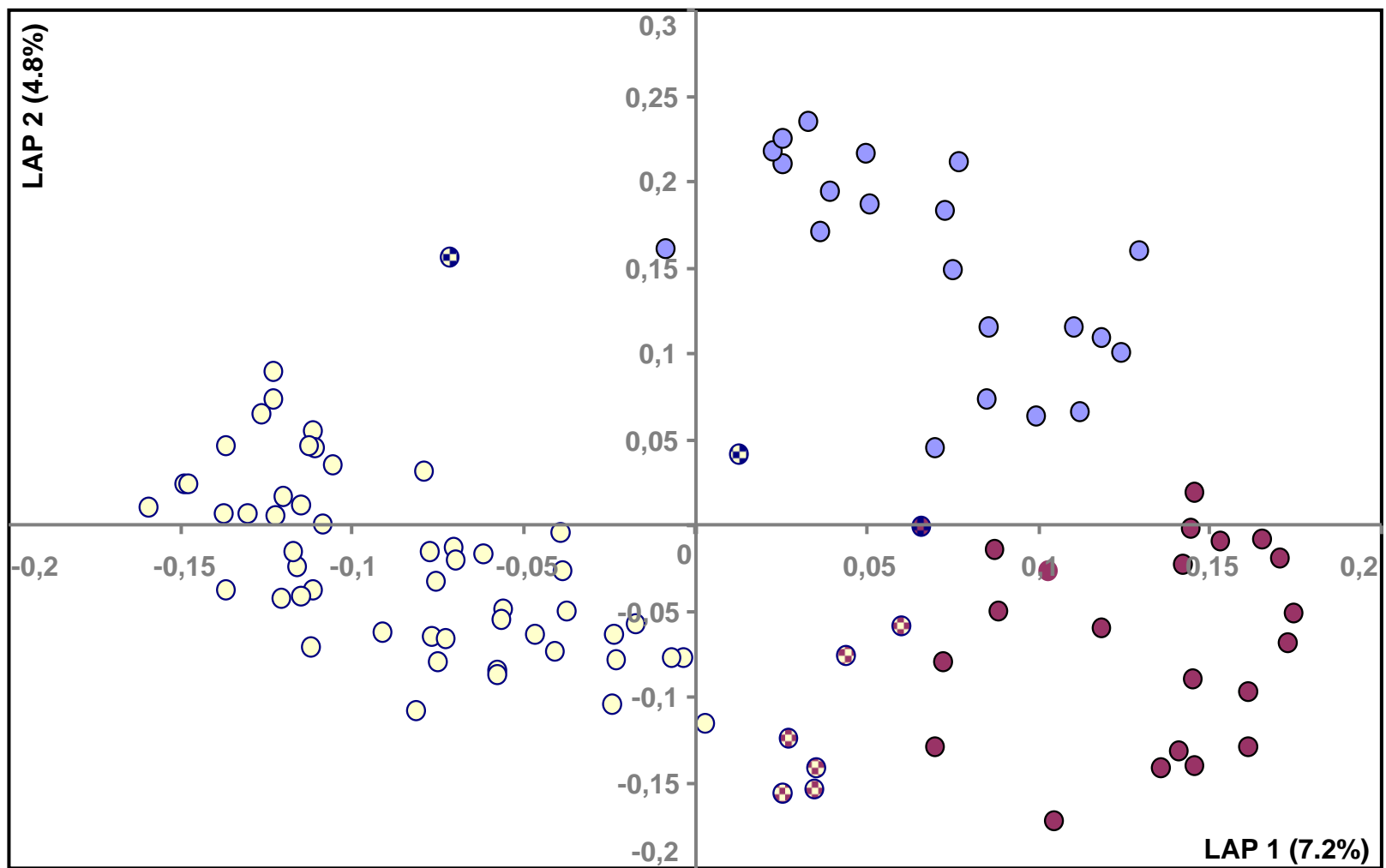

Supplement: Additional file 6 — Laplacian eigenfunction plot based on 230 AFLP loci among glaucous gulls (L. hyperboreus) and North American herring gulls (L. smithsonianus). This file contains the two-dimensional plot of the two first Eigenvectors (and the percentage of the total variance they explain) of LAPEA on 32 Palaearctic hyperboreus, 35 Nearctic hyperboreus, and 35 Nearctic smithsonianus. These LAPEA results correspond with those from STRUCTURE (see figure 5). The three different colours in this LAPEA plot correspond with those in Figure 5. The first Eigenvector clearly separates individuals carrying contributions from the ancestral population indicated by pale yellow from those carrying contributions from the ancestral populations indicated with red and pale blue. The The second Eigenvector separates individuals carrying contributions from the two ancestral populations indicated by pale yellow and red from those carrying contributions from the ancestral population indicated with pale blue. [file 1471-2148-10-348-S6.PDF]
