# Supplementary material for: Introgressive hybridization and the evolutionary history of the herring gull complex revealed by mitochondrial and nuclear DNA
Source: BMC Evol Biol. 2010 Nov 11;10:348. doi: 10.1186/1471-2148-10-348 (PMC2993719; doi:10.1186/1471-2148-10-348)
Supplement: Additional file 3 — Variable positions and Genbank accession numbers of mitochondrial sequences. This file contains a table of aligned variable nucleotide positions of mitochondrial cytochrome b gene and hypervariable region 1 sequences and their Genbank accession numbers for 368 analysed large gulls. The reference sequence is argentatus_0127 from Russia, White Sea. Dotted positions indicate identity to the reverence sequence. Also indicated are taxon membership, geographic origin, and sample ID for each individual. These sequences were used to calculate the median-joining networks in Figure 2 of this article. [file 1471-2148-10-348-S3.PDF]

Additional file 2 – Variable positions and Genbank accession numbers of mitochondrial sequences.

taxon	geographic origin	ID	Acc.no. *CytB*	Acc.no. *HVR1*	*CytB* Gene (59 variable sites)	*HVR1* (40 variable sites)
					0000000000 0000000000 0000000000 0000000000 0000000011 111111111	0000000000 0000000000 0000000000 0000000000
					0011111122 2333333344 4445555555 5666666777 7788889900 000001111	0000000000 0000000000 0111111112 2222222333
					2405667926 8045689922 6890112458 9001237234 5723790302 456780013	2333344445 6666777889 9014488880 0145555235
					4383251984 3784310309 0921365325 8062433466 5182085983 792054503	7346734581 1245057143 5633501474 7284678783
					TGCGATCGTA ATACCTAACT AAATGCACCC TCCATTTATA GTAGACTGAT CAGTTTGTT	CCCGTTTTTT CTAATTCCGC TGGTAGCTCT CCACGAGGGA
argentatus	**Russia, White Sea**	0127	AJ508093	AJ508315	....	....
		0128	FM866243	AJ276950	....C....	....
		0129	FM866244	AJ276947	...A.CC... .T.C...... ....C..... ..G....... ....T.	..CG...T.. .T.A...C.. .....A.... ...C.A....
		0132	AJ508093	AJ508315	....	....
		0133	FM866245	AJ276947	...A.CC... .T.C...... ....G....A.. ....T.	..CG...T.. .T.A...C.. .....A.... ...C.A....
		0218	AJ508093	AJ508315	....	....
		0219	AJ508093	AJ508315	....	....
		2175	AJ508093	AJ276950	....	....
		2176	AJ508099	AJ276950	.... T........	....
		2177	AJ508093	AJ276950	....	....
	**Norway, Tromso**	2119	AJ508093	AJ276950	....	....
		2123	AJ508093	AJ276950	....	....
		2129	AJ508093	AJ508315	....	....
		2120	AJ508101	AJ276947	...A.CC... .T.C...... ....G...C... ....T.	..CG...T.. .T.A...C.. .....A.... ...C.A....
		2122	AJ508101	AJ276947	...A.CC... .T.C...... ....G...C... ....T.	..CG...T.. .T.A...C.. .....A.... ...C.A....
		2130	FM866263	FM866316	...A.CC... .T.C...... ....C..... A.G....... ....T.	..CG...T.. ...A...C.. .....A.... ...C.A....
	**Finland, Lake-Saimaa**	2166	AJ508099	AJ276950	.... T........	....
		2171	AJ508140	AJ508310	.... .T........	..C....... ....CA. ....T. ...C.A..AG
		2172	AJ508093	AJ508325	....	....
		2168	AJ508128	AJ277128	......C... .T.C...G.. ....T.	..CG...T.. .T.A...C.T ....C.A....
		2173	AJ508111	AJ277127	.....CC... .T.C...... ....T.	..CG...T.. .T.A...C.. ....C.A....
		2174	AJ508091	AJ276947	...A.CC... .T.C...... ....T.	..CG...T.. .T.A...C.. .....A.... ...C.A....
	**Estonia, Matsalu**	1208	AJ508093	AJ276950	....	....
		1211	AJ508093	AJ276950	....	....
		1212	AJ508093	AJ276950	....	....
		1209	AJ508091	AJ276946	...A.CC... .T.C...... ....T.	..CG...T.. .T.A...C.. .... T..C.A....
		1210	AJ508128	AJ507815	......C... .T.C...G.. ....T.	..CG...TC. .T.A...C.T ....T. ...C.A....
		1213	AJ508102	AJ277127	...A.CC... .T.C...... ....A...... ....T.	..CG...T.. .T.A...C.. ....C.A....
	**Poland, Wloclawek**	0202	AJ508093	AJ508315	....	....
		0203	AJ508091	AJ277127	...A.CC... .T.C...... ....T.	..CG...T.. .T.A...C.. ....C.A....
	**Germany, Hiddensee**	0902	AJ508093	AJ508315	....	....
		0903	AJ508128	AJ277127	......C... .T.C...G.. ....T.	..CG...T.. .T.A...C.. ....C.A....
		0906	AJ508111	AJ277127	.....CC... .T.C...... ....T.	..CG...T.. .T.A...C.. ....C.A....
		0907	AJ508099	AJ276950	.... T........	....
	**Denmark, Lindholm**	2547	AJ508091	AJ276946		..CG...T.. .T.A...C.. .... T..C.A....
		2548	FM866253	AJ277127	...A.CC... .T.C...... ....G....... ....T.	..CG...T.. .T.A...C.. ....C.A....
		2549	AJ508093	AJ508315	....	....
		2550	AJ508093	AJ508315	....	....
	**Sweden, NW Skane**	2581	AJ508093	AJ276950	....	....
		2583	FM875774	AJ277127	...A.CC... .T.C......	..CG...T.. .T.A...C.. ....C.A....
		2584	FM866269	AJ276950	....T.	....
		2593	AJ508091	FM866319	...A.CC... .T.C...... ....T.	..CG...T.. .T.A...C.. .....A.... ...C.A....
	**Norway, Vest-Agder**	1431	AJ508111	AJ277127	.....CC... .T.C...... ....T.	..CG...T.. .T.A...C.. ....C.A....
		1432	AJ508140	FM866312	.... .T........	..C....... .T.....CA. ....C......
		1445	AJ508093	AJ276950	....	....
		1448	AJ508093	AJ508313	....	.... .A........ ....
		1449	FM866253	AJ276947	...A.CC... .T.C...... ....G....... ....T.	..CG...T.. .T.A...C.. .....A.... ...C.A....
		1450	AJ508102	AJ277127	...A.CC... .T.C...... ....A...... ....T.	..CG...T.. .T.A...C.. ....C.A....
argenteus	**Iceland, Skruder**	2080	AJ508096	AJ508343	...A.CC... .T.C...... ....C..... ....T.	..CG...T.. .T.A...C.. ...C...... ...C.A....
		2081	AJ508093	AJ276950	....	....
		2084	AJ508091	AJ276946	...A.CC... .T.C...... ....T.	..CG...T.. .T.A...C.. .... T..C.A....
		2085	AJ508093	AJ276950	....	....
		2086	AJ508140	AJ508307	.... .T........	..C....... ....CA. ....C......
		2087	AJ508096	AJ508344	...A.CC... .T.C...... ....C..... ....T.	..CG...... .T.A...C.. ....C.A....
		2088	AJ508095	AJ276946	...A.CC... .T.C...... ....G ....T.	..CG...T.. .T.A...C.. .... T..C.A....
		2089	AJ508093	AJ508314	....	.........C ....C..... ..A....... .....A....
		2090	FM866244	AJ276947	...A.CC... .T.C...... ....C..... ..G....... ....T.	..CG...T.. .T.A...C.. .....A.... ...C.A....
		2091	AJ508096	FM866314	...A.CC... .T.C...... ....C..... ....T.	..CG...T.. .T.A...C.. C......... ...C.A....
		2092	AJ508093	AJ508312	....	....T. ....
		2093	FM866261	AJ276947	..TA.CC... .T.C...... ....G....... ....T.	..CG...T.. .T.A...C.. .....A.... ...C.A....
		2094	FM866244	AJ276947	...A.CC... .T.C...... ....C..... ..G....... ....T.	..CG...T.. .T.A...C.. .....A.... ...C.A....
		2095	AJ508093	AJ276950	....	....
		2096	FM866262	AJ277127	...A.CC... .T.C...... ....C... ....T.	..CG...T.. .T.A...C.. ....C.A....
		2099	AJ508140	FM866315	.... .T........	..C....... .T.....C.. ....C......
		2100	AJ508140	FM866315	.... .T........	..C....... .T.....C.. ....C......
		2101	FM866261	AJ276947	..TA.CC... .T.C...... ....G....... ....T.	..CG...T.. .T.A...C.. .....A.... ...C.A....
	**Iceland, Karlsskali**	2102	AJ508093	AJ508312	....	....T. ....
		2105	AJ508136	AJ276950	....C	....
		2106	AJ508094	AJ508311	.... .G........	....A....C.. ..T.......
		2107	FM866244	AJ276947	...A.CC... .T.C...... ....C..... ..G....... ....T.	..CG...T.. .T.A...C.. .....A.... ...C.A....

		2108	FM866244	AJ276947	...A.CC... .T.C...... ....C..... ..G....... ....T.	..CG...T.. .T.A...C.. .....A.... ...C.A....
		2109	AJ508091	AJ276946	...A.CC... .T.C...... ....T.	..CG...T.. .T.A...C.. .... T..C.A....
		2110	AJ508140	FM866308	.... .T........	....CA. ....C......
		2111	AJ508093	AJ276950	....	....
		2112	AJ508136	AJ276950	....C	....
		2114	AJ508091	AJ276946	...A.CC... .T.C...... ....T.	..CG...T.. .T.A...C.. .... T..C.A....
	**Denmark, Faroe Islands**	2035	AJ508096	AJ508344	...A.CC... .T.C...... ....C..... ....T.	..CG...... .T.A...C.. ....C.A....
		2036	AJ508140	AJ508308	.... .T........	..C....... ....C..CA. ....C......
		2037	AJ508096	AJ508344	...A.CC... .T.C...... ....C..... ....T.	..CG...... .T.A...C.. ....C.A....
		2038	AJ508093	AJ508312	....	....T. ....
	**England, Isle of May**	2823	FM866260	AJ277127	...A.CC... .T.C...... ....C..... ....C.T.	..CG...T.. .T.A...C.. ....C.A....
		2827	AJ508091	AJ508344	...A.CC... .T.C...... ....T.	..CG...... .T.A...C.. ....C.A....
		2828	AJ508140	AJ508307	.... .T........	..C....... ....CA. ....C......
		2830	AJ508091	FM866320	...A.CC... .T.C...... ....T.	..CG...... .T.A...C.. ....C.A....
		2831	AJ508140	FM866312	.... .T........	..C....... .T.....CA. ....C......
		2832	AJ508140	FM866315	.... .T........	..C....... .T.....C.. ....C......
	**France, Finistere**	2042	AJ508140	AJ508307	.... .T........	..C....... ....CA. ....C......
		2043	AJ508096	AJ277127	...A.CC... .T.C...... ....C..... ....T.	..CG...T.. .T.A...C.. ....C.A....
		2045	FM866260	AJ277127	...A.CC... .T.C...... ....C..... ....C.T.	..CG...T.. .T.A...C.. ....C.A....
		2054	AJ508091	AJ508344	...A.CC... .T.C...... ....T.	..CG...... .T.A...C.. ....C.A....
		2058	AJ508091	AJ277127	...A.CC... .T.C...... ....T.	..CG...T.. .T.A...C.. ....C.A....
		2044	AJ508143	AJ277130	....G..... .T........ ....T.. ....A..	..C....... ...A...... .... T..CAT....
		2055	AJ508140	AJ508307	.... .T........	..C....... ....CA. ....C......
		2059	AJ508143	AJ277133	....G..... .T........ ....T.. ....A..	..C....... ...A...... .... T..CAT....
	**Netherlands, Maasvlakte**	2180	AJ508091	AJ277127	...A.CC... .T.C...... ....T.	..CG...T.. .T.A...C.. ....C.A....
		2182	AJ508091	AJ277127	...A.CC... .T.C...... ....T.	..CG...T.. .T.A...C.. ....C.A....
		0778	AJ508102	AJ277127	...A.CC... .T.C...... ....A...... ....T.	..CG...T.. .T.A...C.. ....C.A....
		0779	AJ508111	AJ277127	.....CC... .T.C...... ....T.	..CG...T.. .T.A...C.. ....C.A....
		0780	AJ508102	AJ277127	...A.CC... .T.C...... ....A...... ....T.	..CG...T.. .T.A...C.. ....C.A....
		0783	AJ508102	AJ277127	...A.CC... .T.C...... ....A...... ....T.	..CG...T.. .T.A...C.. ....C.A....
		0802	FM866249	FM866307	........C. .T........	..C....... .T.....CA. ....C....A.
		0804	FM866250	AJ276947	...A.CC... .T.C...... ....C..T.	..CG...T.. .T.A...C.. .....A.... ...C.A....
		0805	FM866253	AJ508345	...A.CC... .T.C...... ....G....... ....T.	..CG...T.. ...A...C.. ....C.A....
		0806	AJ508091	FM866308	...A.CC... .T.C...... ....T.	....CA. ....C......
		0781	AJ508102	AJ277127	...A.CC... .T.C...... ....A...... ....T.	..CG...T.. .T.A...C.. ....C.A....
	**Germany, Helgoland**	1394	FM866251	FM866311	.... .T........ .... .T........	.... .T.....C.. ....C......
		1395	FM866252	AJ276950	....T....	....
		1396	AJ508091	AJ277127	...A.CC... .T.C...... ....T.	..CG...T.. .T.A...C.. ....C.A....
		1397	AJ508091	AJ277127	...A.CC... .T.C...... ....T.	..CG...T.. .T.A...C.. ....C.A....
atlantis	**Portugal, Island of Madeira**	1267	AJ508141	AJ277132	.... .T........ ....T..	..C....... ...A.C.... .... T..C.T....
		1268	AJ508141	AJ507766	.... .T........ ....T..	..C....... ....CC.... .... T..C.T....
		1269	AJ508143	AJ508336	....G..... .T........ ....T.. ....A..	T.C.C....C ...A...... .... T..CAT....
		1270	AJ508141	AJ277132	.... .T........ ....T..	..C....... ...A.C.... .... T..C.T....
		1271	AJ508141	AJ507757	.... .T........ ....T..	..C....... ...A.C.... ......T... T..C.T....
	**Morocco, Essaouria**	1639	AJ508141	AJ277132	.... .T........ ....T..	..C....... ...A.C.... .... T..C.T....
		1640	AJ508141	AJ277132	.... .T........ ....T..	..C....... ...A.C.... .... T..C.T....
		1643	AJ508143	AJ277133	....G..... .T........ ....T.. ....A..	..C....... ...A...... .... T..CAT....
		1644	AJ508141	AJ277132	.... .T........ ....T..	..C....... ...A.C.... .... T..C.T....
		1645	AJ508143	AJ277133	....G..... .T........ ....T.. ....A..	..C....... ...A...... .... T..CAT....
	**Portugal, Berlenga Islands**	1613	AJ508143	AJ507762	....G..... .T........ ....T.. ....A..	..C....... ...A...C.. .... T..CAT....
		1616	AJ508143	AJ507762	....G..... .T........ ....T.. ....A..	..C....... ...A...C.. .... T..CAT....
		1617	AJ508143	AJ507762	....G..... .T........ ....T.. ....A..	..C....... ...A...C.. .... T..CAT....
cachinnans	**Rumania, Danube Delta**	0660	AJ508117	AJ507747	......C... .T.C...... ....T.	..CG...TC. .T.A...C.. ....C....C ...C.A....
		0661	AJ508117	AJ508337	......C... .T.C...... ....T.	..CG...T.C .T.A...C.. ....C....C ...C.A....
		0662	AJ508119	AJ507758	......C... .T.C...... ......G... ...G...... ....G..... ....T.	....C..T.. .T.A...C.. ....C.A..A.
		0663	AJ508117	AJ507746	......C... .T.C...... ....T.	..CG...T.C .T.A...C.. ....C....C ...C.A....
		0664	AJ508119	AJ507759	......C... .T.C...... ......G... ...G...... ....G..... ....T.	....C..T.. .T.A...C.. ....A..A.
		0665	AJ508119	AJ507761	......C... .T.C...... ......G... ...G...... ....G..... ....T.	....C..T.C .T.A...C.. ....A..A.
	**Ukraine, Odessa**	1653	AJ508117	AJ507749	......C... .T.C...... ....T.	..CG...TC. .T.A...C.T ....C....C ...C.A....
		1655	AJ508117	AJ507750	......C... .T.C...... ....T.	..CG...TCC .T.A...C.. ....C....C ...C.A....
		1656	AJ508117	AJ507746	......C... .T.C...... ....T.	..CG...T.C .T.A...C.. ....C....C ...C.A....
		1657	AJ508116	AJ277127	.....CC... .T.C...... .... .C........ ....T.	..CG...T.. .T.A...C.. ....C.A....
	**Ukraine, Azov' Black Sea**	0745	AJ508117	AJ507750	......C... .T.C...... ....T.	..CG...TCC .T.A...C.. ....C....C ...C.A....
		0746	AJ508117	AJ508338	......C... .T.C...... ....T.	..CG...TC. .T.A...C.. ....C....C ...C.A....
		0747	FM866248	AJ508338	.....CC... .T.C...... ....T.	..CG...TC. .T.A...C.. ....C....C ...C.A....
		0748	AJ508117	AJ277134	......C... .T.C...... ....T.	..CG...T.. .T.A...C.. ....C....C ...C.A....
		0750	AJ508111	AJ277127	......C... .T.C...... ....T.	..CG...T.. .T.A...C.. ....C.A....
		0751	AJ508117	AJ508338	......C... .T.C...... ....T.	..CG...TC. .T.A...C.. ....C....C ...C.A....
		0752	AJ508117	AJ277134	......C... .T.C...... ....T.	..CG...T.. .T.A...C.. ....C....C ...C.A....
	**Russia, N Caspian Sea**	1094	AJ508111	AJ277127	.....CC... .T.C...... ....T.	..CG...T.. .T.A...C.. ....C.A....
		1095	AJ508117	FM866310	......C... .T.C...... ....T.	..CG...TC. TT.A...C.. ....C....C ...C.A....
		1096	AJ508117	AJ277134	......C... .T.C...... ....T.	..CG...T.. .T.A...C.. ....C....C ...C.A....
		1097	AJ508117	AJ508338	......C... .T.C...... ....T.	..CG...TC. .T.A...C.. ....C....C ...C.A....
		1098	AJ508117	AJ277134	......C... .T.C...... ....T.	..CG...T.. .T.A...C.. ....C....C ...C.A....
		1099	AJ508117	AJ508338	......C... .T.C...... ....T.	..CG...TC. .T.A...C.. ....C....C ...C.A....
		1117	AJ508114	AJ276942	......C... .T.C...... ....T C......... ....T.	..CG...TC. .T.A...C.. ....C....C ...C.A....
		1118	AJ508117	AJ507748	......C... .T.C...... ....T.	..CG...TC. .T.A...C.. ....C....C ...CAA....
		1119	AJ508117	AJ508339	......C... .T.C...... ....T.	..CG...TC. .T.A...C.T ....C....C ...C.A....

		1124	AJ508117	AJ277134	......C... .T.C...... ....T.	..CG...T.. .T.A...C.. ....C....C ...C.A....
		1125	AJ508117	AJ508335	......C... .T.C...... ....T.	..CG...TC. .T.A...C.. ....C....C ...CAA....
		1126	AJ508115	AJ507759	......C... .T.C...... ......G.T. ...G...... ....G..... ....T.	....C..T.. .T.A...C.. ....A..A.
		1127	AJ508119	AJ507759	......C... .T.C...... ......G... ...G...... ....G..... ....T.	....C..T.. .T.A...C.. ....A..A.
		1128	AJ508119	AJ507759	......C... .T.C...... ......G... ...G...... ....G..... ....T.	....C..T.. .T.A...C.. ....A..A.
		1129	AJ508117	AJ276942	......C... .T.C...... ....T.	..CG...TC. .T.A...C.. ....C....C ...C.A....
		1130	AJ508117	AJ276942	......C... .T.C...... ....T.	..CG...TC. .T.A...C.. ....C....C ...C.A....
fuscus	**Finland, Lake Saimaa**	2207	AJ508128	AJ277128	......C... .T.C...G.. ....T.	..CG...T.. .T.A...C.T ....C.A....
		2208	AJ508128	AJ277128	......C... .T.C...G.. ....T.	..CG...T.. .T.A...C.T ....C.A....
		2209	AJ508122	AJ277127	......C... .T.C...G.. ....C..T.	..CG...T.. .T.A...C.. ....C.A....
		2210	AJ508122	AJ277127	......C... .T.C...G.. ....C..T.	..CG...T.. .T.A...C.. ....C.A....
		2221	AJ508128	AJ277127	......C... .T.C...G.. ....T.	..CG...T.. .T.A...C.. ....C.A....
		2222	AJ508128	AJ277127	......C... .T.C...G.. ....T.	..CG...T.. .T.A...C.. ....C.A....
		1484	AJ508117	AJ508342	......C... .T.C...... ....T.	..CG...T.. .T.A...C.. ....C.A..A.
		1485	AJ508128	AJ507813	......C... .T.C...G.. ....T.	..CG...T.. .T.....C.T ....C......
		1486	AJ508128	AJ277127	......C... .T.C...G.. ....T.	..CG...T.. .T.A...C.. ....C.A....
		1487	AJ508123	AJ277127	......C... .T.C...G.. ..G....... ....T.	..CG...T.. .T.A...C.. ....C.A....
		1488	AJ508123	AJ277127	......C... .T.C...G.. ..G....... ....T.	..CG...T.. .T.A...C.. ....C.A....
		2643	AJ508128	AJ277128	......C... .T.C...G.. ....T.	..CG...T.. .T.A...C.T ....C.A....
		2644	AJ508128	AJ277127	......C... .T.C...G.. ....T.	..CG...T.. .T.A...C.. ....C.A....
		2645	AJ508128	AJ277127	......C... .T.C...G.. ....T.	..CG...T.. .T.A...C.. ....C.A....
		2646	AJ508128	AJ277127	......C... .T.C...G.. ....T.	..CG...T.. .T.A...C.. ....C.A....
		2647	AJ508124	AJ508324	C.....C... .T.C.C.G.. ....T.	..CG..CT.. .T.....C.. ....C.A....
		0142	FM866246	AJ277127	.A....C... .T.C...G.. ....T.	..CG...T.. .T.A...C.. ....C.A....
graellsi	**Denmark, Faeroe Islands**	1920	AJ508125	AJ277127	......C... .T.C...G.. ....G..... ....T.	..CG...T.. .T.A...C.. ....C.A....
		1921	AJ508125	AJ277128	......C... .T.C...G.. ....G..... ....T.	..CG...T.. .T.A...C.T ....C.A....
		1922	AJ508125	AJ507744	......C... .T.C...G.. ....G..... ....T.	..CG...T.. .T.....C.T ....C.A....
		1923	AJ508128	AJ277128	......C... .T.C...G.. ....T.	..CG...T.. .T.A...C.T ....C.A....
	**Iceland**	1664	AJ508127	AJ277127	......CA.. .T.C...G.. ....T.	..CG...T.. .T.A...C.. ....C.A....
		1665	AJ508126	AJ277128	......C... .T.C..GG.. ....T.	..CG...T.. .T.A...C.T ....C.A....
		1666	AJ508125	AJ277128	......C... .T.C...G.. ....G..... ....T.	..CG...T.. .T.A...C.T ....C.A....
		1667	AJ508127	AJ277128	......CA.. .T.C...G.. ....T.	..CG...T.. .T.A...C.T ....C.A....
		1668	AJ508127	AJ507741	......CA.. .T.C...G.. ....T.	..CG...T.. .T.A...C.T .A........ ...C.A....
		1669	AJ508128	AJ277128	......C... .T.C...G.. ....T.	..CG...T.. .T.A...C.T ....C.A....
		1670	AJ508128	AJ507744	......C... .T.C...G.. ....T.	..CG...T.. .T.....C.T ....C.A....
		1673	AJ508128	AJ507742	......C... .T.C...G.. ....T.	..CG...T.. .T.A..TC.T ....C.A....
hyperboreus Eura	**Russia, Novaja Semlja**	0669	AJ508093	AJ508322	....	....T... ....C.. ....
		0913	AJ508093	AJ276950	....	....
		0914	AJ508093	AJ508316	....	...G...... ....
		0915	AJ508138	AJ276950	....G...	....
		0916	AJ508138	AJ276950	....G...	....
		0917	AJ508099	AJ276950	.... T........	....
		0918	AJ508099	AJ276950	.... T........	....
		0919	AJ508138	AJ276950	....G...	....
	**Svalbard, Longyearbyen**	1902	AJ508139	AJ508323	C	....G....... ....C......
		1903	AJ508093	AJ276950	....	....
		1904	AJ508093	AJ508320	....	.... .A........ ...C......
		1906	AJ508093	AJ276950	....	....
		1907	AJ508093	AJ276950	....	....
		1908	AJ508093	AJ508321	....	....A. ....C......
		1909	AJ508093	AJ508316	....	...G...... ....
		1910	AJ508093	AJ276950	....	....
		1911	AJ508093	AJ508318	....	....C......
		1912	AJ508093	AJ508318	....	....C......
	**Denmark, Faroe Islands**	0939	AJ508093	AJ508323	....	....G....... ....C......
		0940	AJ508093	AJ276950	....	....
		0941	AJ508093	AJ508316	....	...G...... ....
		0942	AJ508148	AJ276939	......C..G .T.C...... ....T.	..CG...T.. .T.A...C.. ....CAA..A.
		1013	AJ508133	AJ276938	......C..G .TGC...... ....T.	..CG...T.. .T.A...C.. ....CAA....
	**Iceland, Bjarnhafnarfjall**	1866	AJ508136	AJ276950	....C	....
		1867	AJ508093	AJ276950	....	....
		1868	AJ508136	AJ276950	....C	....
		1869	AJ508136	AJ508317	....C	.... .T........ ....
		1870	AJ508136	AJ276950	....C	....
		1871	AJ508136	AJ276950	....C	....
		1872	AJ508093	AJ276950	....	....
		1874	AJ508136	AJ276950	....C	....
		1875	AJ508136	AJ276950	....C	....
yperboreus N. America	**Canada, NWT, Baffin Island**	1842	FM875773	AJ276940	......C..G .T.C...... ....C ....C..T.	..CG...T.. .T.A...C.. ....CAA..AG
		1843	AJ508135	AJ276940	......C..G .T.C...... ....C ....T.	..CG...T.. .T.A...C.. ....CAA..AG
		1845	AJ508107	AJ277127	......C..G .T.C...T.. ....T.	..CG...T.. .T.A...C.. ....C.A....
		1846	FM866255	AJ277129	......C..G .T.C...... G......... ....T.	..CG...T.. .T.A...C.. ....CAA..AG
		1847	FM866256	AJ276940	......C..G .T.C...... ....C ......AT.	..CG...T.. .T.A...C.. ....CAA..AG
		1848	AJ508107	AJ277127	......C..G .T.C...T.. ....T.	..CG...T.. .T.A...C.. ....C.A....
		1849	FM866257	AJ507821	......C..G .T.C...G.. ....T.	..CG...T.. .T.A...C.. C......... ...CAA..AG
		1850	AJ508148	AJ277129	......C..G .T.C...... ....T.	..CG...T.. .T.A...C.. ....CAA..AG
		1851	FM866258	AJ276939	......C..G .T.C...... ....A..... ....T.	..CG...T.. .T.A...C.. ....CAA..A.
		1852	AJ508148	AJ277129	......C..G .T.C...... ....T.	..CG...T.. .T.A...C.. ....CAA..AG

h		1854	AJ508148	AJ277129	......C..G .T.C...... ....T.	..CG...T.. .T.A...C.. ....CAA..AG
		1855	AJ508148	AJ277129	......C..G .T.C...... ....T.	..CG...T.. .T.A...C.. ....CAA..AG
		1856	AJ508148	AJ276939	......C..G .T.C...... ....T.	..CG...T.. .T.A...C.. ....CAA..A.
		1861	AJ508148	AJ277129	......C..G .T.C...... ....T.	..CG...T.. .T.A...C.. ....CAA..AG
		1862	AJ508135	AJ507822	......C..G .T.C...... ....C ....T.	..C....T.. .T.A...C.. ....CAA..AG
		1864	AJ508148	AJ277129	......C..G .T.C...... ....T.	..CG...T.. .T.A...C.. ....CAA..AG
		1865	AJ508133	AJ276938	......C..G .TGC...... ....T.	..CG...T.. .T.A...C.. ....CAA....
	**USA, Alaska, Yukon**	3014	AJ508148	FM866327	......C..G .T.C...... ....T.	..CG.C.T.. .T.A...C.. ....CAA..AG
		3015	AJ508135	AJ276940	......C..G .T.C...... ....C ....T.	..CG...T.. .T.A...C.. ....CAA..AG
		3016	AJ508109	AJ276939	......C..G .T.C...... ....T....... ....T.	..CG...T.. .T.A...C.. ....CAA..A.
		3017	AJ508135	AJ276940	......C..G .T.C...... ....C ....T.	..CG...T.. .T.A...C.. ....CAA..AG
		3018	AJ508148	FM866328	......C..G .T.C...... ....T.	..CG...T.. .T.....C.T ....CAA..AG
		3019	AJ508135	FM866329	......C..G .T.C...... ....C ....T.	..CG...T.. .T.A...C.. ....CAA..AG
		3020	AJ508148	AJ507821	......C..G .T.C...... ....T.	..CG...T.. .T.A...C.. C......... ...CAA..AG
	**USA, NW Alaska**	3140	AJ508148	FM866329	......C..G .T.C...... ....T.	..CG...T.. .T.A...C.. ....CAA..AG
		3141	AJ508135	FM866329	......C..G .T.C...... ....C ....T.	..CG...T.. .T.A...C.. ....CAA..AG
		3142	AJ508148	AJ276938	......C..G .T.C...... ....T.	..CG...T.. .T.A...C.. ....CAA....
		3143	AJ508148	FM866330	......C..G .T.C...... ....T.	..CG...T.. .T.....C.T ....CAA..A.
		3144	AJ508148	FM866329	......C..G .T.C...... ....T.	..CG...T.. .T.A...C.. ....CAA..AG
		3146	AJ508135	FM866329	......C..G .T.C...... ....C ....T.	..CG...T.. .T.A...C.. ....CAA..AG
		3147	AJ508133	FM866331	......C..G .TGC...... ....T.	..CG...TC. .T.A...C.. ....CAA..A.
		3148	AJ508148	FM866332	......C..G .T.C...... ....T.	..CG...T.. .T.A...C.. C......... ...CAA..AG
		3149	AJ508135	FM866329	......C..G .T.C...... ....C ....T.	..CG...T.. .T.A...C.. ....CAA..AG
	**USA, Washington**	3154	AJ508133	AJ276940	......C..G .TGC...... ....T.	..CG...T.. .T.A...C.. ....CAA..AG
		3155	AJ508148	FM866333	......C..G .T.C...... ....T.	.TCG...T.. .T.A...C.. ....CAA..AG
***marinus* Europe**	**Denmark, Katholm, Jylland**	0043	AJ508140	FM866306	.... .T........	..C....T.. ....C.. ....T. ...C.A..A.
		0908	AJ508140	AJ276949	.... .T........	..C....... ....CA. ....T. ...C......
		0946	AJ508140	AJ276949	.... .T........	..C....... ....CA. ....T. ...C......
		0962	AJ508140	AJ276948	.... .T........	..C....T.. ....CA. ....T. ...C.A..A.
		0963	AJ508140	AJ276949	.... .T........	..C....... ....CA. ....T. ...C......
		0964	AJ508140	FM866309	.... .T........	..C....... ....CA. ....T. ...C....A.
		2525	FM866265	AJ276948	.... .T........ ....T.	..C....T.. ....CA. ....T. ...C.A..A.
		2527	AJ508140	AJ276948	.... .T........	..C....T.. ....CA. ....T. ...C.A..A.
		2528	FM866266	AJ276948	.... .T........ ....C ....T.	..C....T.. ....CA. ....T. ...C.A..A.
		2529	FM866265	FM866309	.... .T........ ....T.	..C....... ....CA. ....T. ...C....A.
		2530	AJ508140	AJ276948	.... .T........	..C....T.. ....CA. ....T. ...C.A..A.
		2533	AJ508140	FM866309	.... .T........	..C....... ....CA. ....T. ...C....A.
	**Netherlands, Maasvlakte**	2233	AJ508140	FM866318	.... .T........	..C....T.. ....CA. ....T. ...C.A..A.
		2234	AJ508140	FM866309	.... .T........	..C....... ....CA. ....T. ...C....A.
	**France, Finistere**	2159	AJ508140	AJ276948	.... .T........	..C....T.. ....CA. ....T. ...C.A..A.
		2160	AJ508140	FM866317	.... .T........	T.C.C..... ....CA. ....T. ...C....A.
	**Denmark, Faroe Islands**	0993	AJ508140	AJ276948	.... .T........	..C....T.. ....CA. ....T. ...C.A..A.
		0994	AJ508140	AJ276948	.... .T........	..C....T.. ....CA. ....T. ...C.A..A.
		0996	AJ508140	AJ276949	.... .T........	..C....... ....CA. ....T. ...C......
		0997	AJ508140	AJ508306	.... .T........	..C....... ....CA. ....T. ...CA.....
		0998	AJ508140	AJ276948	.... .T........	..C....T.. ....CA. ....T. ...C.A..A.
		0999	AJ508140	AJ508304	.... .T........	..C....T.. ......TCA. ....T. ...C.A..A.
		1000	AJ508140	AJ276949	.... .T........	..C....... ....CA. ....T. ...C......
		1001	AJ508140	AJ508305	.... .T........	..CG...... ....CA. ....T. ...CA.....
		1002	AJ508148	AJ276939	......C..G .T.C...... ....T.	..CG...T.. .T.A...C.. ....CAA..A.
		1003	AJ508140	AJ508304	.... .T........	..C....T.. ......TCA. ....T. ...C.A..A.
		1004	AJ508140	FM866309	.... .T........	..C....... ....CA. ....T. ...C....A.
		1021	AJ508140	AJ276948	.... .T........	..C....T.. ....CA. ....T. ...C.A..A.
		1022	AJ508140	AJ276949	.... .T........	..C....... ....CA. ....T. ...C......
		1024	AJ508140	AJ276949	.... .T........	..C....... ....CA. ....T. ...C......
	**Iceland, Skruder**	2161	AJ508140	AJ276949	.... .T........	..C....... ....CA. ....T. ...C......
		2162	AJ508140	AJ276949	.... .T........	..C....... ....CA. ....T. ...C......
***marinus* N. America**	**Canada, Newfoundland**	2163	AJ508107	AJ276938	......C..G .T.C....T. ....T.	..CG...T.. .T.A...C.. ....CAA....
		2164	FM866264	FM866309	.... .T........ ...C...... ....T.	..C....... ....CA. ....T. ...C....A.
		2165	AJ508107	AJ277127	......C..G .T.C....T. ....T.	..CG...T.. .T.A...C.. ....C.A....
	**Canada, Bay of Fundy**	2905	AJ508140	AJ276949	.... .T........	..C....... ....CA. ....T. ...C......
		2906	AJ508140	AJ276949	.... .T........	..C....... ....CA. ....T. ...C......
		2907	AJ508140	AJ276949	.... .T........	..C....... ....CA. ....T. ...C......
		2908	AJ508140	FM866322	.... .T........	..C....... ....CA. ....T. ...C......
		2909	FM866270	FM866309	.... .T........ ...C......	..C....... ....CA. ....T. ...C....A.
		2910	AJ508107	AJ508344	......C..G .T.C....T. ....T.	..CG...... .T.A...C.. ....C.A....
		2911	FM866270	FM866309	.... .T........ ...C......	..C....... ....CA. ....T. ...C....A.
		2912	AJ508140	AJ276949	.... .T........	..C....... ....CA. ....T. ...C......
		2913	AJ508140	AJ276949	.... .T........	..C....... ....CA. ....T. ...C......
		2914	FM866270	FM866323	.... .T........ ...C......	..C....... ..G....CA. ....T. ...C....A.
		2915	AJ508107	FM866324	......C..G .T.C....T. ....T.	..CG...T.. .T.A...C.. ....C.. ...C.A....
	**Canada, Lake Ontario**	2898	FM866270	FM866321	.... .T........ ...C......	..CG...T.. ....CA. ....T. ...C....A.
		2900	AJ508107	AJ277127	......C..G .T.C....T. ....T.	..CG...T.. .T.A...C.. ....C.A....
		2901	AJ508140	AJ276949	.... .T........	..C....... ....CA. ....T. ...C......
		2902	AJ508107	AJ508344	......C..G .T.C....T. ....T.	..CG...... .T.A...C.. ....C.A....
		2903	AJ508140	AJ276949	.... .T........	..C....... ....CA. ....T. ...C......
		2904	AJ508107	AJ277127	......C..G .T.C....T. ....T.	..CG...T.. .T.A...C.. ....C.A....

		2921	FM866270	FM866325	.... .T........ ...C......	..C....... ....CA. ....C....A.
		2922	AJ508140	AJ276949	.... .T........	..C....... ....CA. ....T. ...C......
		2923	AJ508107	AJ277127	......C..G .T.C....T. ....T.	..CG...T.. .T.A...C.. ....C.A....
		2924	AJ508107	AJ277127	......C..G .T.C....T. ....T.	..CG...T.. .T.A...C.. ....C.A....
		2927	AJ508140	AJ276949	.... .T........	..C....... ....CA. ....T. ...C......
		2928	AJ508107	AJ277127	......C..G .T.C....T. ....T.	..CG...T.. .T.A...C.. ....C.A....
		2930	AJ508140	AJ276949	.... .T........	..C....... ....CA. ....T. ...C......
		2931	AJ508140	FM866326	.... .T........	..C....... ....C.. ....T. ...C......
		2932	FM866265	AJ276949	.... .T........ ....T.	..C....... ....CA. ....T. ...C......
		2933	AJ508140	AJ277127	.... .T........	..CG...T.. .T.A...C.. ....C.A....
		2936	AJ508140	AJ276949	.... .T........	..C....... ....CA. ....T. ...C......
		2937	AJ508140	AJ276949	.... .T........	..C....... ....CA. ....T. ...C......
michahelli	**Greece, Island of Crete**	0772	AJ508143	AJ507754	....G..... .T........ ....T.. ....A..	..C....... ...A..T... .... T..CAT..A.
		0773	AJ508143	AJ507756	....G..... .T........ ....T.. ....A..	..C....... ...A..T... .... TA.CAT....
		0774	AJ508143	AJ507765	....G..... .T........ ....T.. ....A..	..C......C ...A...... .... T..CAT....
		0775	AJ508143	AJ277131	....G..... .T........ ....T.. ....A..	..C....... ...A..T... .... T..CAT....
		0655	AJ508143	AJ277131	....G..... .T........ ....T.. ....A..	..C....... ...A..T... .... T..CAT....
	**Italy, Capraia Island**	0034	AJ508143	AJ277131	....G..... .T........ ....T.. ....A..	..C....... ...A..T... .... T..CAT....
		0035	AJ508143	AJ507752	....G..... .T........ ....T.. ....A..	..C....... ...A...... .... T..CATA...
		0036	AJ508143	AJ277130	....G..... .T........ ....T.. ....A..	..C....... ...A...... .... T..CAT....
		0037	AJ508143	AJ277131	....G..... .T........ ....T.. ....A..	..C....... ...A..T... .... T..CAT....
	**Malta, Filfla Island**	1200	AJ508143	AJ277131	....G..... .T........ ....T.. ....A..	..C....... ...A..T... .... T..CAT....
		1202	AJ508143	AJ277133	....G..... .T........ ....T.. ....A..	..C....... ...A...... .... T..CAT....
	**France, Alsace**	1381	AJ508143	AJ507763	....G..... .T........ ....T.. ....A..	..C....... ...A...... ....C.. T..CAT....
		1383	AJ508143	AJ507763	....G..... .T........ ....T.. ....A..	..C....... ...A...... ....C.. T..CAT....
	**Spain, Gibraltar**	1627	AJ508145	AJ277133	....G..... .T..T..... ....T.. ....A..	..C....... ...A...... .... T..CAT....
		1628	AJ508145	AJ277133	....G..... .T..T..... ....T.. ....A..	..C....... ...A...... .... T..CAT....
		1636	AJ508143	AJ277130	....G..... .T........ ....T.. ....A..	..C....... ...A...... .... T..CAT....
		1637	AJ508143	AJ277131	....G..... .T........ ....T.. ....A..	..C....... ...A..T... .... T..CAT....
		1638	AJ508144	AJ277131	....G..... .T........ ....T.. ....A.. .G.......	..C....... ...A..T... .... T..CAT....
smithsoniana	**Canada, Lake Ontario**	0287	AJ508107	AJ277127	......C..G .T.C....T. ....T.	..CG...T.. .T.A...C.. ....C.A....
		0289	AJ508107	AJ277127	......C..G .T.C....T. ....T.	..CG...T.. .T.A...C.. ....C.A....
		0290	AJ508107	AJ277127	......C..G .T.C....T. ....T.	..CG...T.. .T.A...C.. ....C.A....
		0291	AJ508104	AJ277127	......C..G .T.C....T. ....G. ....T.	..CG...T.. .T.A...C.. ....C.A....
		0292	AJ508133	AJ276938	......C..G .TGC...... ....T.	..CG...T.. .T.A...C.. ....CAA....
		0293	AJ508106	AJ276938	......C..G .TGC...... ....C...T.	..CG...T.. .T.A...C.. ....CAA....
	**Canada, New Brunsewick**	1999	FM866259	AJ277127	......C..G GT.C....T. ....G. ....T.	..CG...T.. .T.A...C.. ....C.A....
		2000	AJ508107	AJ277127	......C..G .T.C....T. ....T.	..CG...T.. .T.A...C.. ....C.A....
		2001	AJ508107	AJ507745	......C..G .T.C....T. ....T.	..CG...T.C .T.A...C.. ....C.A....
		2002	AJ508107	FM866313	......C..G .T.C....T. ....T.	..CG...T.. .T.A...C.. ....C.A.A..
		2004	AJ508107	AJ277127	......C..G .T.C....T. ....T.	..CG...T.. .T.A...C.. ....C.A....
		2005	AJ508107	AJ277127	......C..G .T.C....T. ....T.	..CG...T.. .T.A...C.. ....C.A....
		2010	AJ508107	AJ277127	......C..G .T.C....T. ....T.	..CG...T.. .T.A...C.. ....C.A....
		2013	AJ508107	AJ277127	......C..G .T.C....T. ....T.	..CG...T.. .T.A...C.. ....C.A....
		2014	AJ508103	AJ277127	......C..G .T.C....T. ....A....T.	..CG...T.. .T.A...C.. ....C.A....
		2015	AJ508107	AJ277127	......C..G .T.C....T. ....T.	..CG...T.. .T.A...C.. ....C.A....
		2016	AJ508103	AJ277127	......C..G .T.C....T. ....A....T.	..CG...T.. .T.A...C.. ....C.A....
		2017	AJ508107	AJ276938	......C..G .T.C....T. ....T.	..CG...T.. .T.A...C.. ....CAA....
		2018	AJ508107	AJ276938	......C..G .T.C....T. ....T.	..CG...T.. .T.A...C.. ....CAA....
		2020	AJ508107	AJ277127	......C..G .T.C....T. ....T.	..CG...T.. .T.A...C.. ....C.A....
		2003	-	AJ277127		..CG...T.. .T.A...C.. ....C.A....
	**Canada, Prince Edward Island**	2022	AJ508107	AJ277127	......C..G .T.C....T. ....T.	..CG...T.. .T.A...C.. ....C.A....
		2023	AJ508107	AJ277127	......C..G .T.C....T. ....T.	..CG...T.. .T.A...C.. ....C.A....
		2024	AJ508107	AJ277127	......C..G .T.C....T. ....T.	..CG...T.. .T.A...C.. ....C.A....
		2025	AJ508107	AJ277127	......C..G .T.C....T. ....T.	..CG...T.. .T.A...C.. ....C.A....
		2026	AJ508107	AJ277127	......C..G .T.C....T. ....T.	..CG...T.. .T.A...C.. ....C.A....
		2027	AJ508107	AJ277127	......C..G .T.C....T. ....T.	..CG...T.. .T.A...C.. ....C.A....
		2028	AJ508107	AJ277127	......C..G .T.C....T. ....T.	..CG...T.. .T.A...C.. ....C.A....
		2029	AJ508107	AJ277127	......C..G .T.C....T. ....T.	..CG...T.. .T.A...C.. ....C.A....
		2030	AJ508107	AJ276946	......C..G .T.C....T. ....T.	..CG...T.. .T.A...C.. .... T..C.A....
	**USA, Alaska, Fearbanks**	2141	AJ508148	AJ507817	......C..G .T.C...... ....T.	..CG...T.. .T.....C.. ....CAA..A.
		2142	AJ508108	AJ507818	......C..G .T.C...... ....T.... ....T.	..CG...T.. .T.A...C.. ....CAA..A.
		2143	AJ508109	AJ276940	......C..G .T.C...... ....T....... ....T.	..CG...T.. .T.A...C.. ....CAA..AG
		2144	AJ508148	AJ276939	......C..G .T.C...... ....T.	..CG...T.. .T.A...C.. ....CAA..A.
		2145	AJ508107	AJ277127	......C..G .T.C....T. ....T.	..CG...T.. .T.A...C.. ....C.A....
